# Supplementary material for: 4-O-Glucosylation of Trichothecenes by Fusarium Species: A Phase II Xenobiotic Metabolism for t-Type Trichothecene Producers
Source: Int J Mol Sci. 2021 Dec 17;22(24):13542. doi: 10.3390/ijms222413542 (PMC8709292; doi:10.3390/ijms222413542)
Supplement: Supplementary file 1 [file ijms-22-13542-s001.zip › ijms-1505767-supplementary.pdf]

Supplementary Materials

4-*O*-glucosylation of trichothecenes by *Fusar*ium species: a phase II xenobiotic metabolism for t-type trichothecene producers

Kosuke Matsui[1], Hirone Takeda[2], Koki Shinkai[2], Takao Kakinuma[3], Yoshiaki Koizumi[2], Masahiro Kase[2], Tomoya Yoshinari[4], Hiroaki Minegishi[2], Yuichi Nakajima[1], Shunichi Aikawa[5], Naoko Takahashi-Ando[2,3,5,*], Makoto Kimura[1]

[1]Graduate School of Bioagricultural Sciences, Nagoya University, Furo-cho, Chikusa-ku, Nagoya, Aichi 464-8601, Japan; matsuik.toyo@gmail.com (K.M.); nakajima.yuichi@a.mbox.nagoya-u.ac.jp (Y.N.); mkimura@agr.nagoya-u.ac.jp (M. Kimura)

[2]Graduate School of Science and Engineering, Toyo University, 2100 Kujirai, Kawagoe, Saitama 350-8585, Japan; s36d02000102@toyo.jp (H.T.); s16d01301215@gmail.com (K.S.); s36d02000045@toyo.jp (Y.K.); s36D02100079@toyo.jp (M. Kase); minehiro@toyo.jp (H.M.); ando_n@toyo.jp (N.T-A.)

[3]Faculty of Science and Engineering, Toyo University, 2100 Kujirai, Kawagoe, Saitama 350-8585, Japan; tt19970815@gmail.com (T.K.)

[4]Division of Microbiology, National Institute of Health and Sciences, Kanagawa 210-9501, Japan; yoshinari@nihs.go.jp (T.Y.)

[5]Research Institute of Industrial Technology, Toyo University, 2100 Kujirai, Kawagoe, Saitama 350-8585, Japan; s-aikawa@toyo.jp (S.A.)

*Correspondence: Naoko Takahashi-Ando

ando_n@toyo.jp; +81-49-239-1384

Table S1. NMR spectroscopic data for **1** and **2**.

TDmol-glc (**1**)			
position		δ_H (ppm)	δ_C (ppm)
2	CH	3.69 (d, 5.4)	80.5
3	CH_a	2.51 (dd, 15.2, 8.0)	38.8
3	CH_b	2.00 (ddd, 15.2, 5.4, 3.7)	
4	CH	4.49 (dd, 8.0, 3.7)	79.4
5	C	-	51.0
6	C	-	41.2
7	CH_a	1.88 (dt, 12.6, 5.7)	25.3
7	CH_b	1.47 (ddt, 12.6, 5.4, 1.7)	
8	CH_2	1.90-2.10 (m)	29.0
9	C	-	141.3
10	CH	5.35 (brd, 4.9)	119.8
11	CH	3.58-3.63** (m)	71.8
12	C	-	67.4
13	CH_a	3.11 (d, 4.0)	49.1***
13	CH_b	2.95 (d, 4.0)	
14	CH_3	0.85 (s)	6.8
15	CH_3	0.91 (s)	16.4
16	CH_3	1.70 (brs)	23.3
1'	CH	4.86* (m)	100.8
2'	CH	3.36 (dd, 9.7, 3.7)	74.1
3'	CH	3.61-3.89** (m)	75.0
4'	CH	3.24 (t, 9.3)	72.1
5'	CH	3.61-3.89** (m)	74.4
6'	CH_a	3.86 (brd, 9.7)	62.9
6'	CH_b	3.61-3.89** (m)	

TCC-glc (**2**)			
position		δ_H (ppm)	δ_C (ppm)
2	CH	3.83 (d, 5.2)	80.9
3	CH_a	2.60 (dd, 15.5, 7.9)	38.9
3	CH_b	2.15 (ddd, 15.5, 5.2, 3.9)	
4	CH	4.46 (dd, 7.9, 3.9)	78.8
5	C	-	51.0
6	C	-	44.5
7	CH_a	2.87 (dd, 15.4, 0.9)	43.0
7	CH_b	2.26 (d, 15.4)	
8	CH_2	-	201.0
9	C=O	-	138.6
10	CH	6.53 (brd, 5.9)	139.5
11	CH	3.98 (brd, 5.9)	71.1
12	C	-	67.3
13	CH_a	3.14 (d, 3.7)	48.6
13	CH_b	2.98 (d, 3.7)	
14	CH_3	0.85 (s)	6.5
15	CH_3	1.00 (d, 0.9)	18.7
16	CH_3	1.77 (brs)	15.3
1'	CH	4.86 (d, 3.7)	101.0
2'	CH	3.36 (dd, 9.7, 3.7)	74.0
3'	CH	3.61-3.70**** (m)	74.8
4'	CH	3.25 (t, 9.3)	72.1
5'	CH	3.61-3.70**** (m)	74.4
6'	CH_a	3.84-3.89 (m)	62.9
6'	CH_b	3.61-3.70**** (m)	

*Overlapped peak of CH and H_2O.
**Overlapped peak of CH and three kinds of CH on the structure of sugar.
***Overlapped peak of C and solvent.
****Overlapped peak of three kinds of CH on the structure of sugar.

Table S2. NMR spectroscopic data for **3**.

HT-2-glc (**3**)			
position		δ_H (ppm)	δ_C (ppm)
2	CH	3.49 (d, 4.9)	80.6
3	CH	4.28 (dd, 4.9, 3.2)	79.1
4	CH	4.57 (d, 3.2)	87.8
5	C	-	50.5
6	C	-	44.1
7	CH_a	1.89 (brd, 15.4)	29.1
7	CH_b	2.40 (dd,15.4, 5.7)	
8	CH	5.32 (d,5.7)	69.5
9	C	-	137.2
10	CH	5.75 (brd, 5.7)	125.2
11	CH	4.27 (brd, 5.7)	68.5
12	C	-	66.1****
13	CH_a	2.89 (d, 4.0)	48.2
13	CH_b	3.04 (d, 4.0)	
14	CH_3	0.86 (s)	7.5
15	CH_a	3.99 (d, 12.6)	66.1****
15	CH_b	4.42 (d,12.6)	
16	CH_3	1.74 (brs)	20.4
1'	C=O	-	172.2
2'	CH_a	2.17 (dd, 14.6, 6.6)	44.5
2'	CH_b	2.21 (dd, 14.6, 7.7)	
3'	CH	2.04-2.14* (m)	26.9
4' / 5'	CH_3	0.98 (d, 6.6)	22.8
5' / 4'	CH_3	0.97 (d, 6.6)	22.7
1"	C=O	-	174.0
2"	CH_3	2.08 (s)	21.1
1'''	CH	4.91 (d, 4.0)	101.3
2'''	CH	3.41 (dd, 10.0, 4.0)	74.0
3'''	CH	3.61-3.71** (m)	75.0
4'''	CH	3.26-3.33*** (m)	72.0
5'''	CH	3.79 (ddd, 9.9, 6.4, 2.0)	74.3
6'''	CH_a	3.61-3.71** (m)	62.6
6'''	CH_b	3.91 (dd, 11.5, 2.0)	

*Overlapped peak of CH and CH_3.
**Overlapped peak of two kinds of CH on the structure of sugar.
***Overlapped peak of CH and solvent.
****Overlapped peak of two kinds of carbon.

Table S3. NMR spectroscopic data for **4**.

THA-glc (**4**)			
position		δ_H (ppm)	δ_C (ppm)
2	CH	3.98 (dd, 11.7, 6.3)	79.0
3	CH_a	1.81 (q, 11.7)	40.2
3	CH_b	2.44-2.53 (m)	
4	CH	3.89 (dd, 11.7, 5.4)	80.7
5	C	-	60.2
6	C	-	44.5
7	CH_a	1.31 (dd, 12.6, 5.7)	26.8
7	CH_b	1.67 (dd, 12.6, 6.0)	
8	CH_a	1.92 (dd, 18.3, 5.7)	29.3
8	CH_b	2.02-3.13 (m)	
9	C	-	141.9
10	CH	5.50 (dm, 4.3)	119.5
11	CH	3.70-3.74* (m)	78.6
12	C	-	91.5
13	CH_a	3.71 (d,11.7)	65.6
13	CH_b	3.78 (d,11.7)	
14	CH_3	1.10 (s)	10.6
15	CH_3	0.89 (s)	15.0
16	CH_3	1.71 (brs)	23.8
1'	CH	4.87 (d,3.7)	101.9
2'	CH	3.36 (dd, 9.7, 3.7)	74.0
3'	CH	3.63 (dd, 9.7, 8.9)	75.2
4'	CH	3.29-3.34** (m)	72.2
5'	CH	3.59-3.64*** (m)	74.4
6'	CH_a	3.69 (dd, 12.0, 5.2)	62.9
6'	CH_b	3.77 (d, 12.0, 2.6)	

*Overlapped peak of two kinds of CH.
**Overlapped peak of CH and solvent.
***Overlapped peak of two kinds of CH on the structure of sugar.

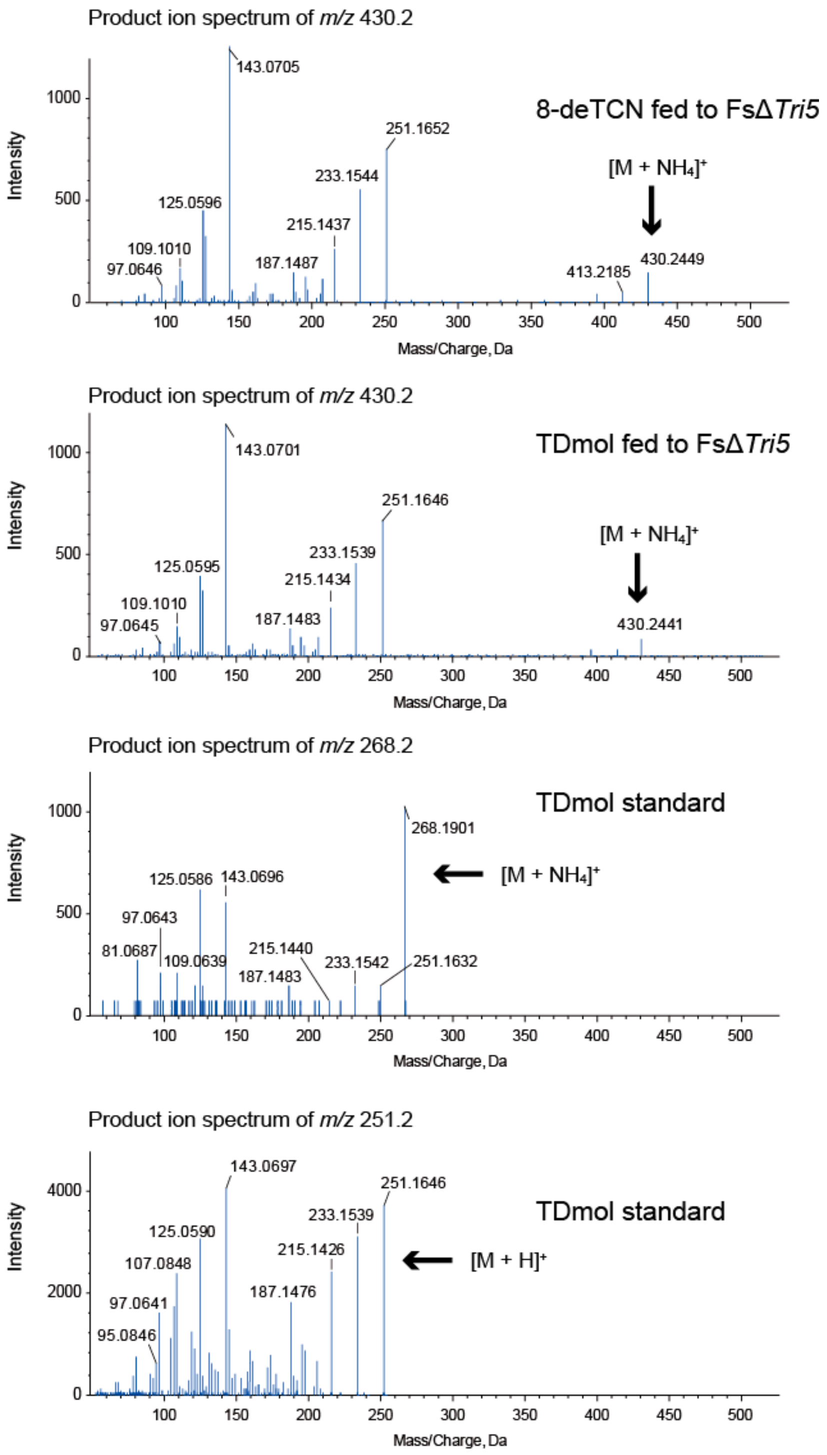

Figure S1. LC-MS/MS analysis of the acetonitrile extract of the culture fed with trichodermol (TDmol) and 8-deoxytrichothecin (8-deTCN). The trichothecenes fed to the culture of strain FsΔ*Tri5* were recovered at 48 h.

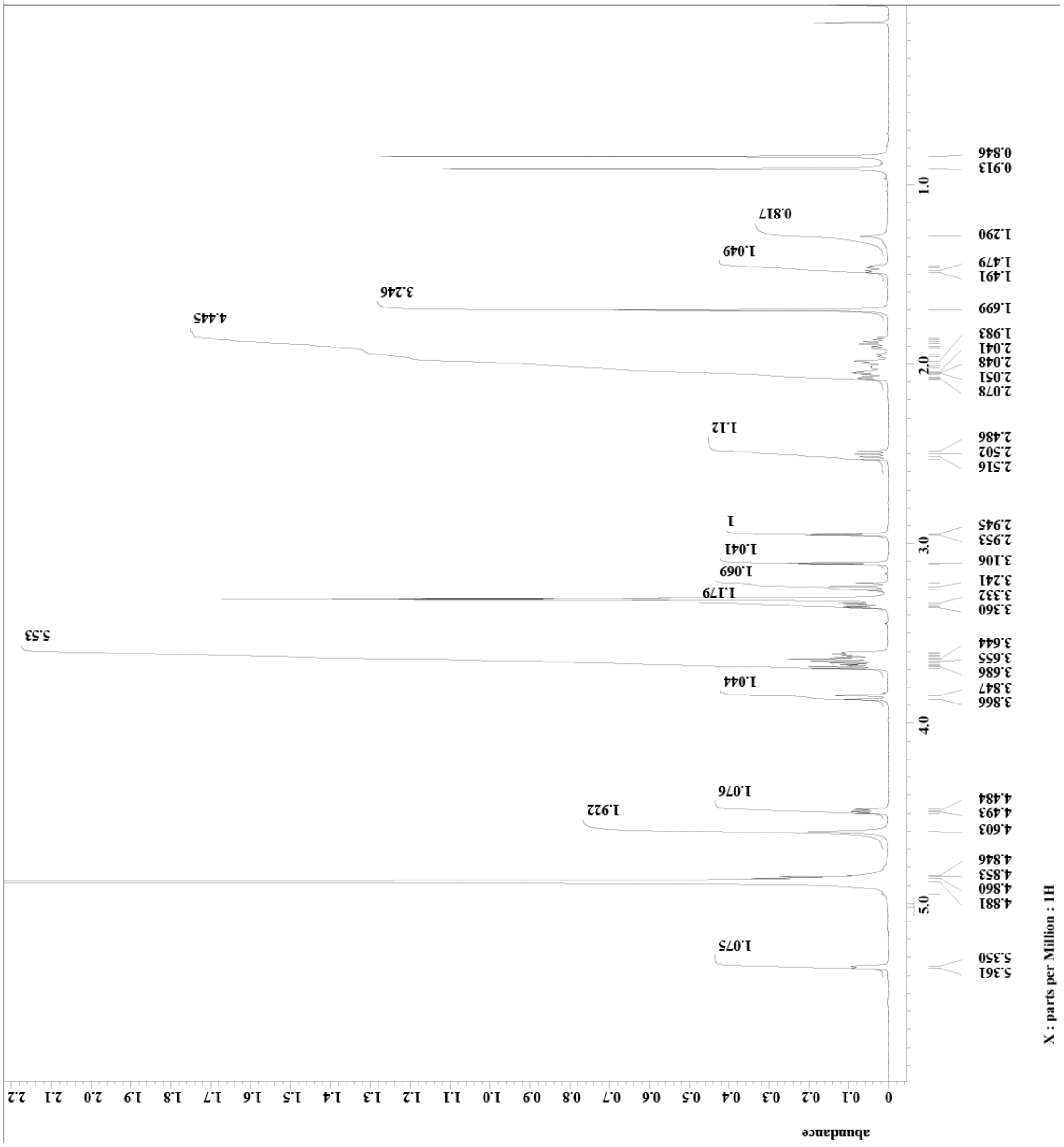

Figure S2-a. ^{1}H-NMR spectrum of **1**.

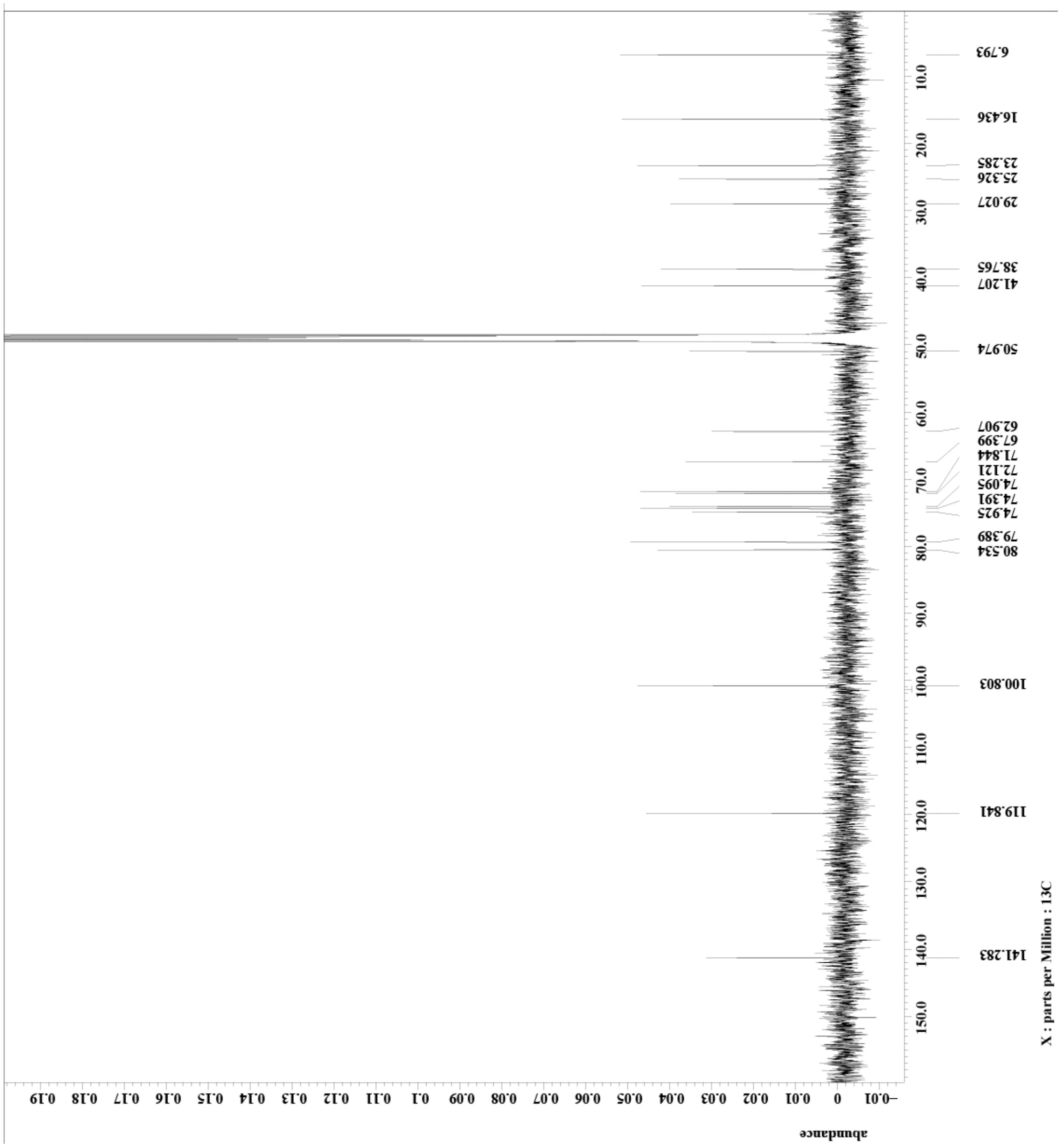

Figure S2-b. ^{13}C NMR spectrum of **1**.

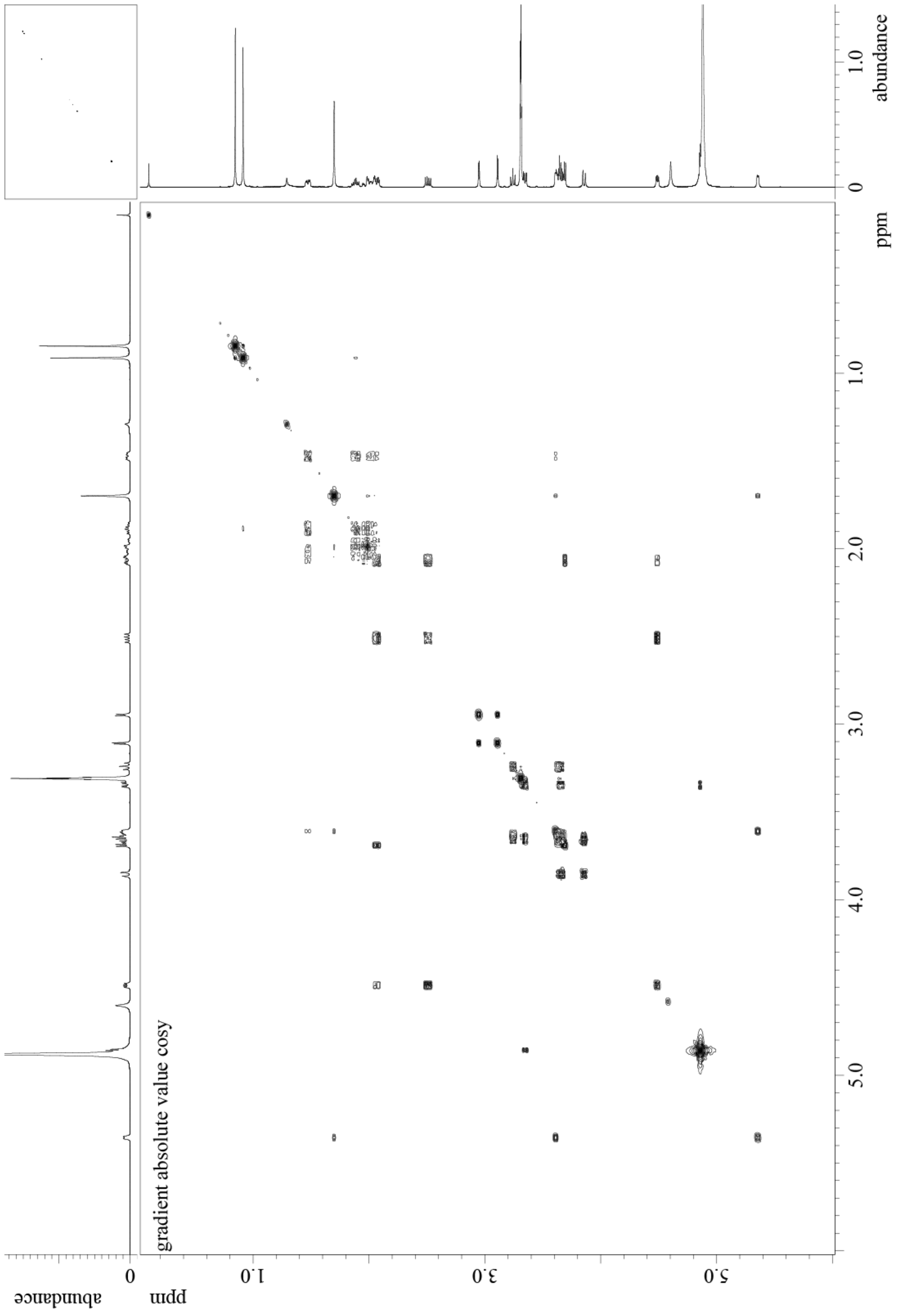

Figure S2-c. Correlation spectroscopy (COSY) spectrum of **1**.

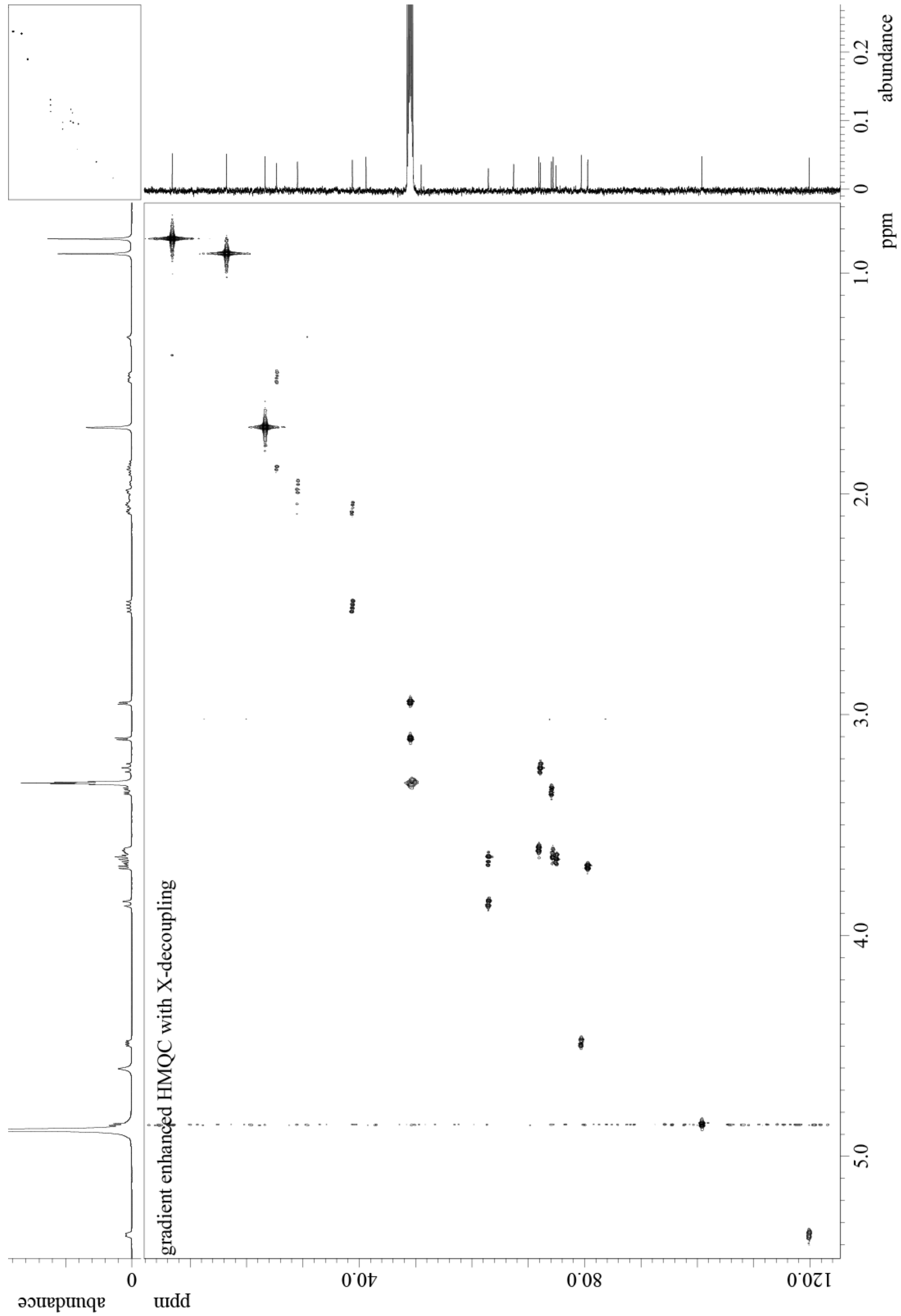

Figure S2-d. Heteronuclear multiple quantum coherence (HMQC) spectrum of **1**.

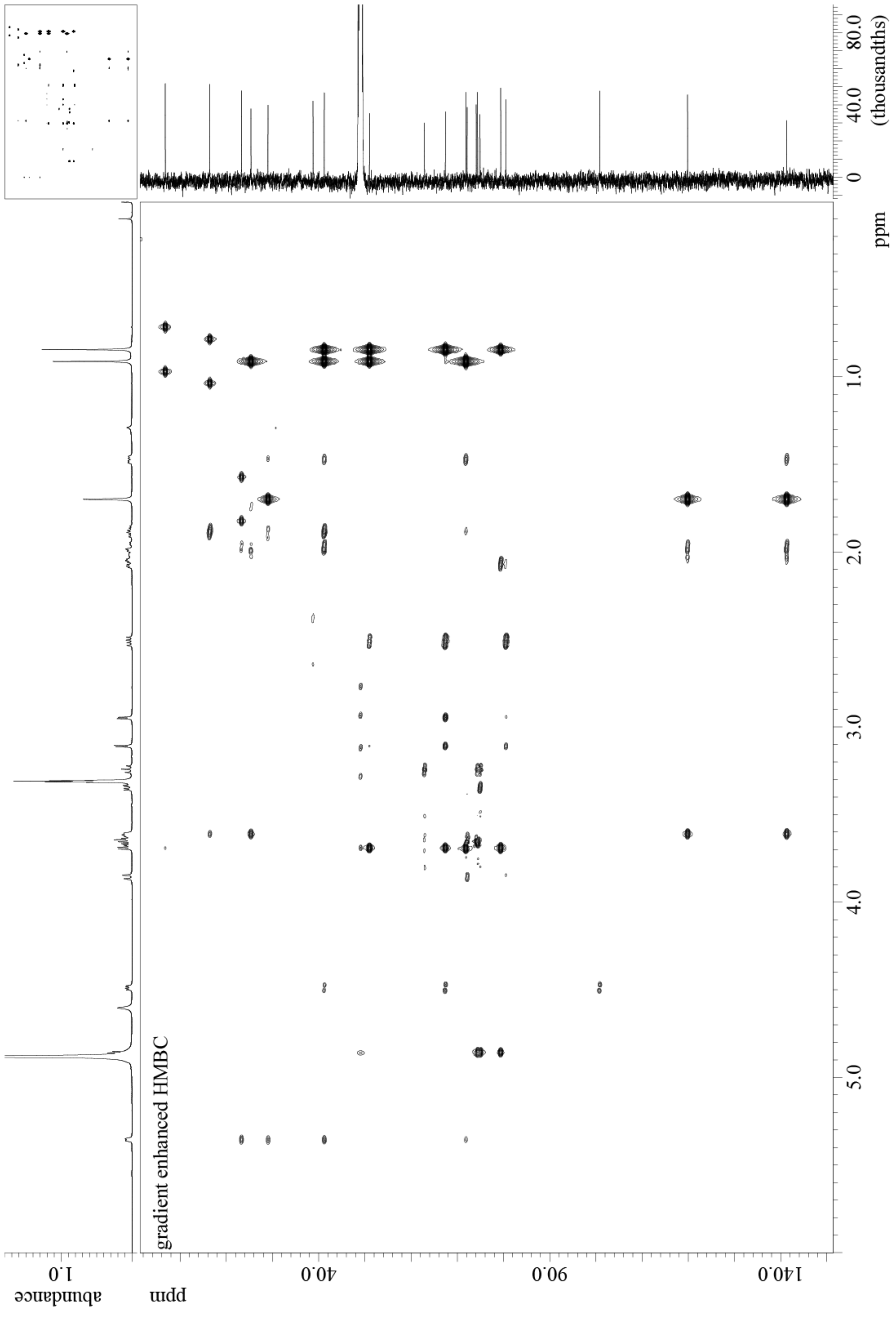

Figure S2-e. Heteronuclear multiple bond coherence (HMBC) spectrum of **1**.

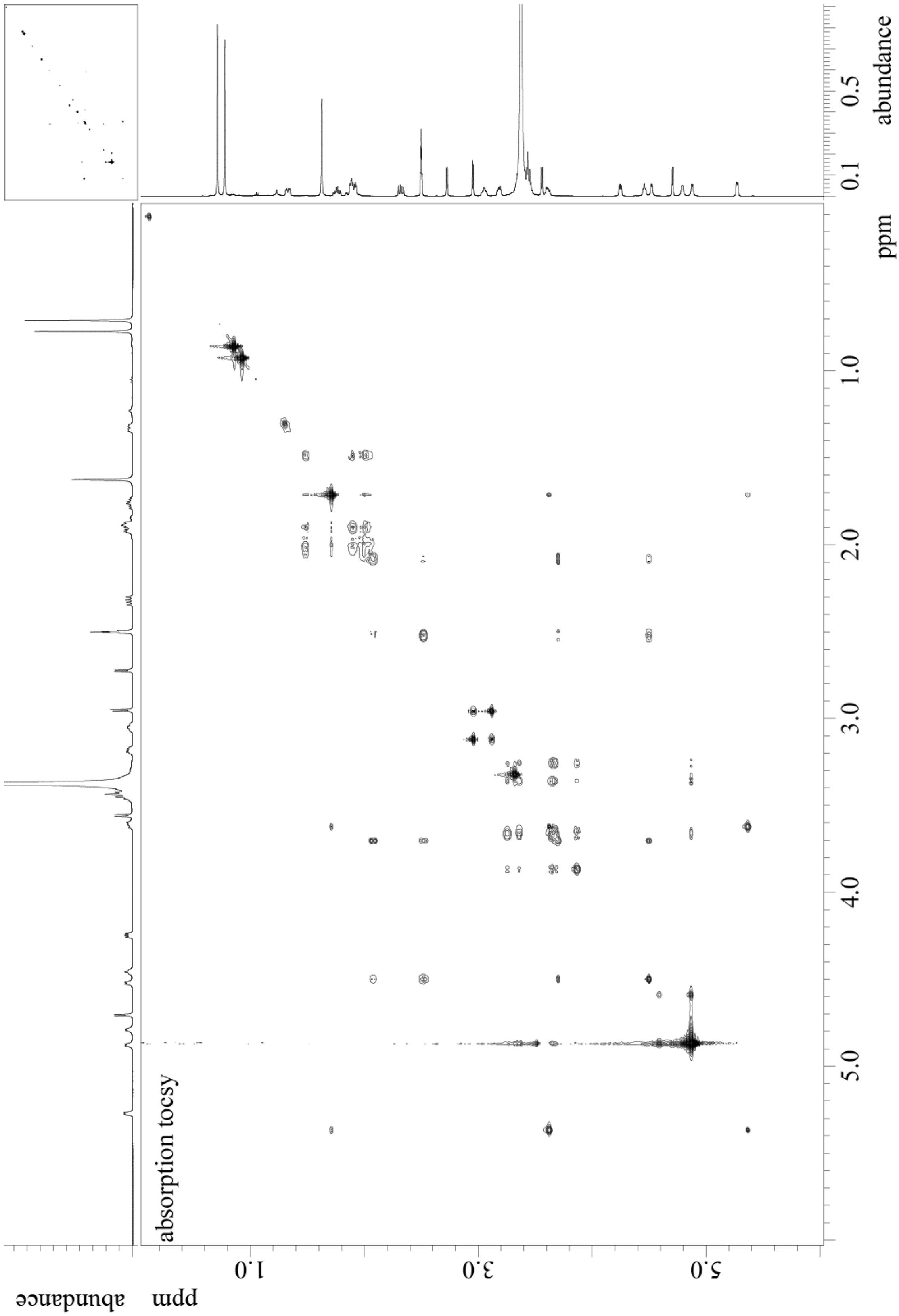

Figure S2-f. Total correlation spectroscopy (TOCSY) spectrum of **1**.

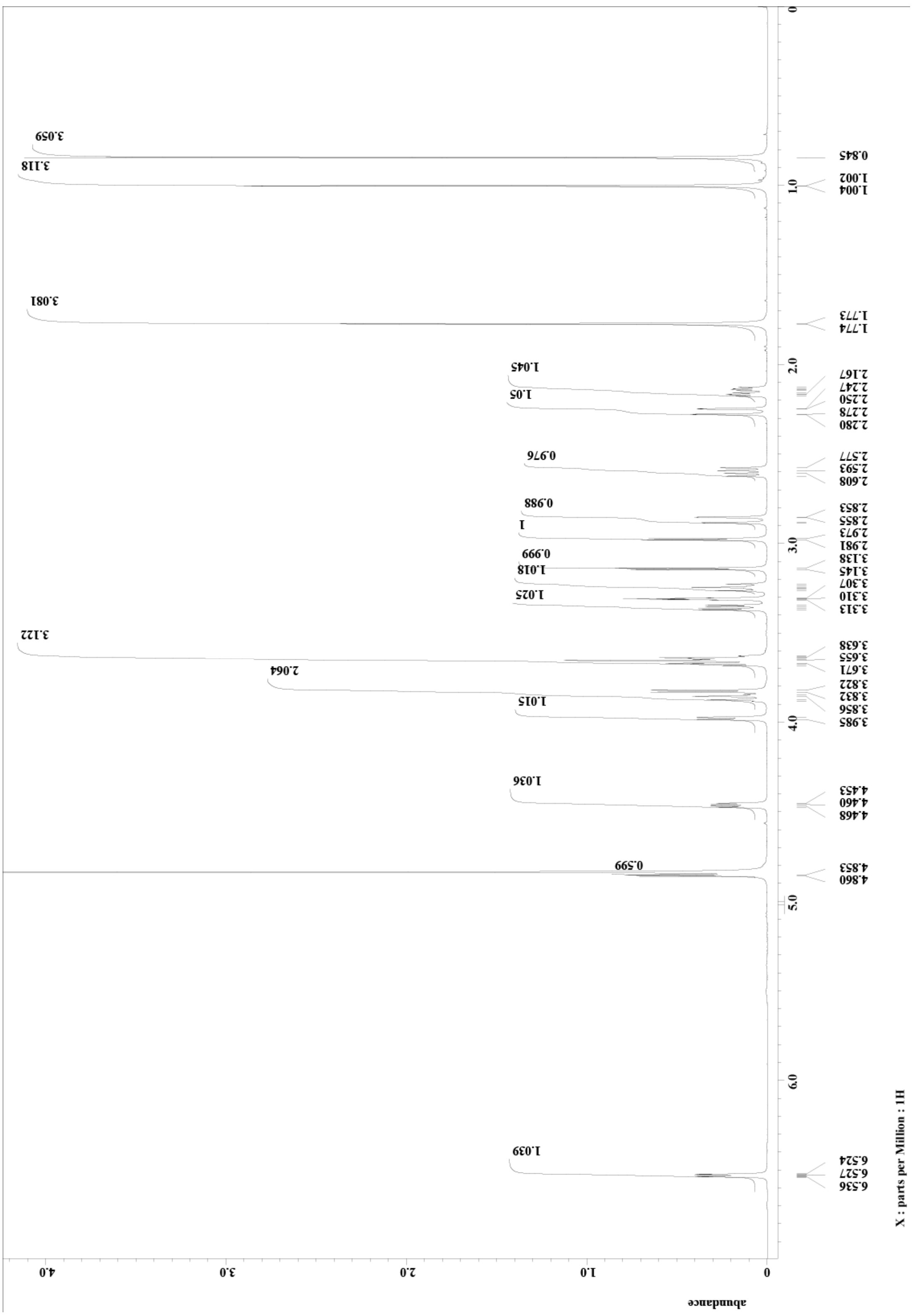

Figure S3-a. ^{1}H NMR spectrum of **2**.

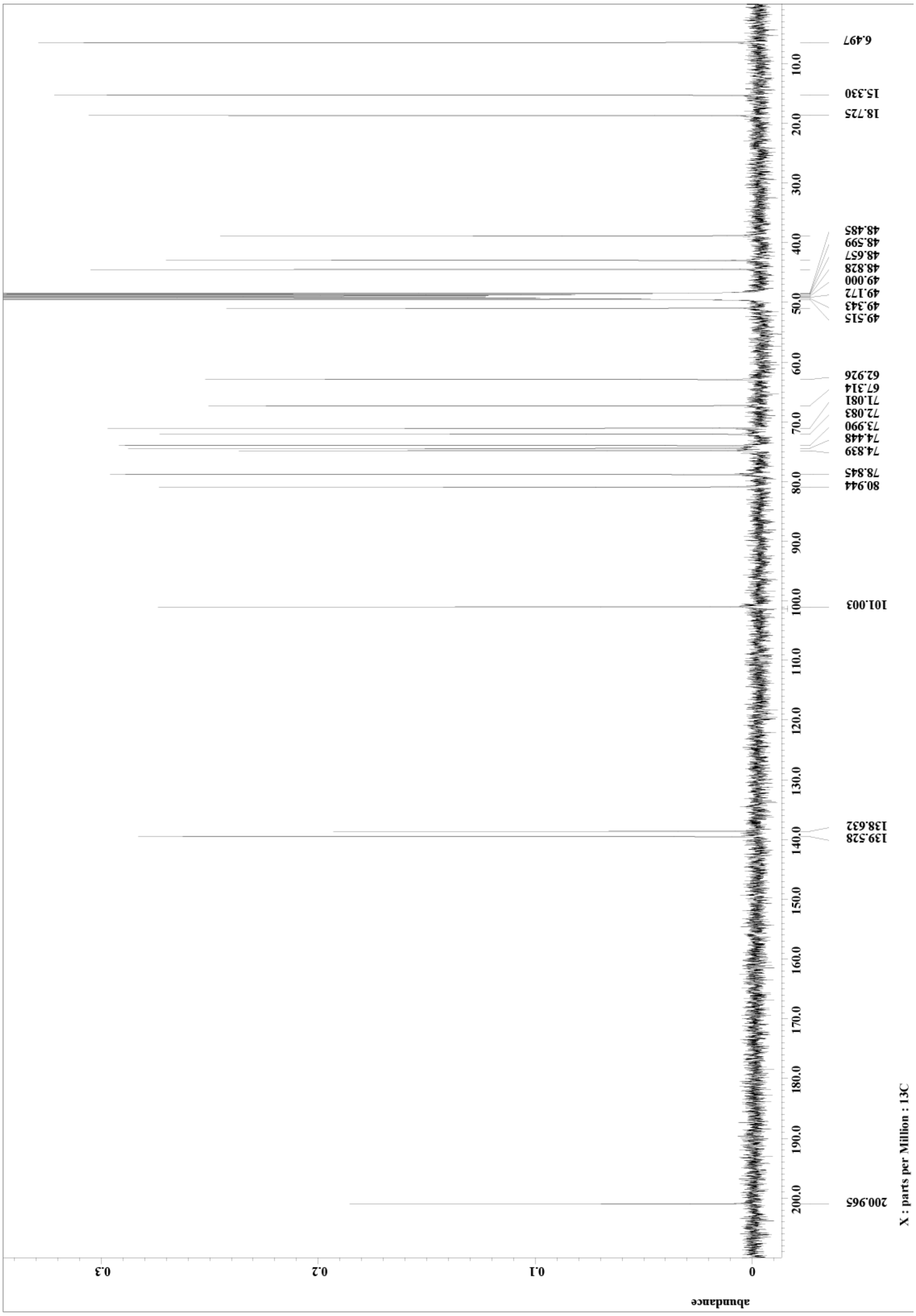

Figure S3-b. ^{13}C NMR spectrum of **2**.

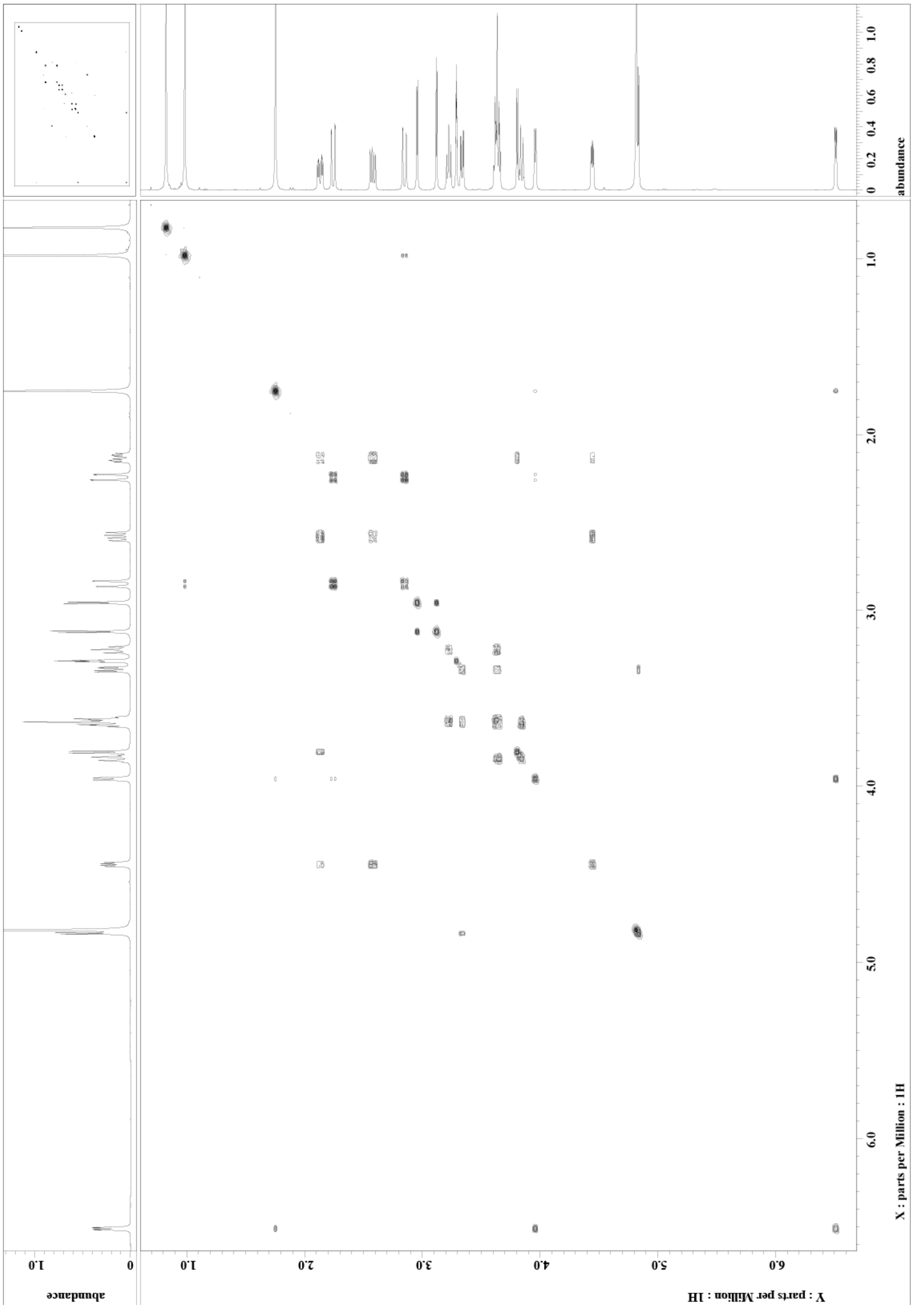

Figure S3-c. Correlation spectroscopy (COSY) spectrum of **2**.

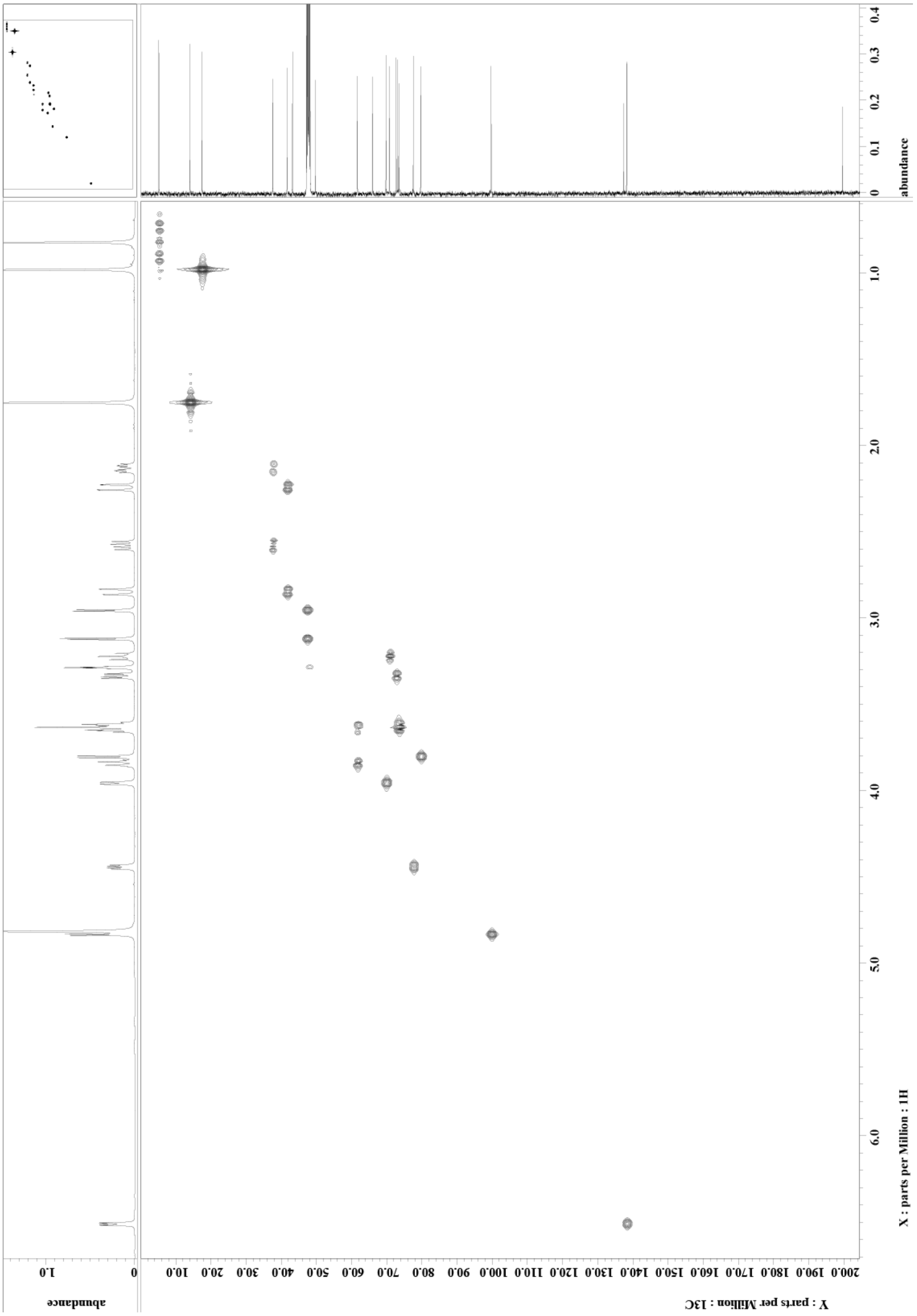

Figure S3-d. Heteronuclear multiple quantum coherence (HMQC) spectrum of **2**.

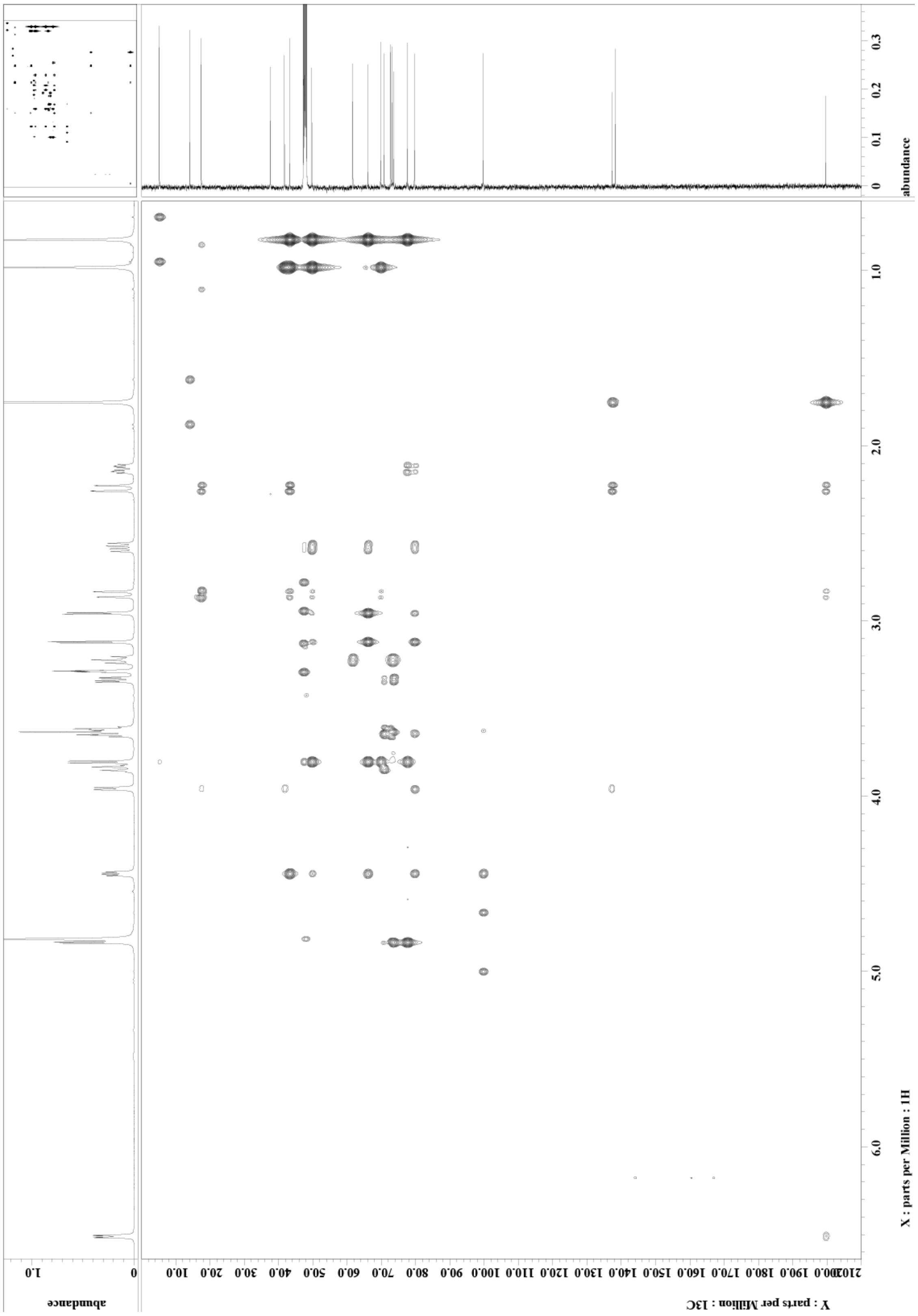

Figure S3-e. Heteronuclear multiple bond coherence (HMBC) spectrum of **2**.

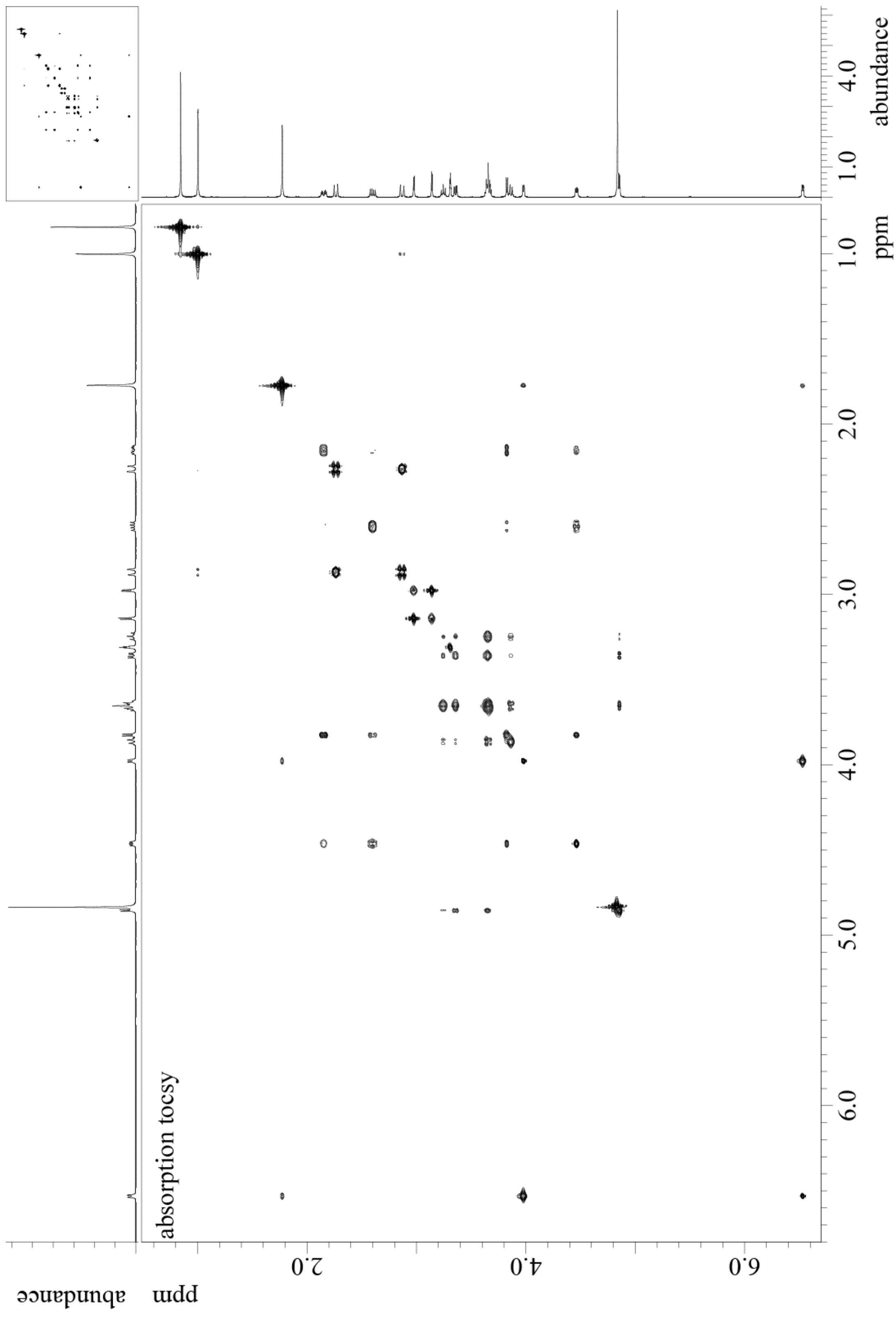

Figure S3-f. Total correlation spectroscopy (TOCSY) spectrum of **2**.

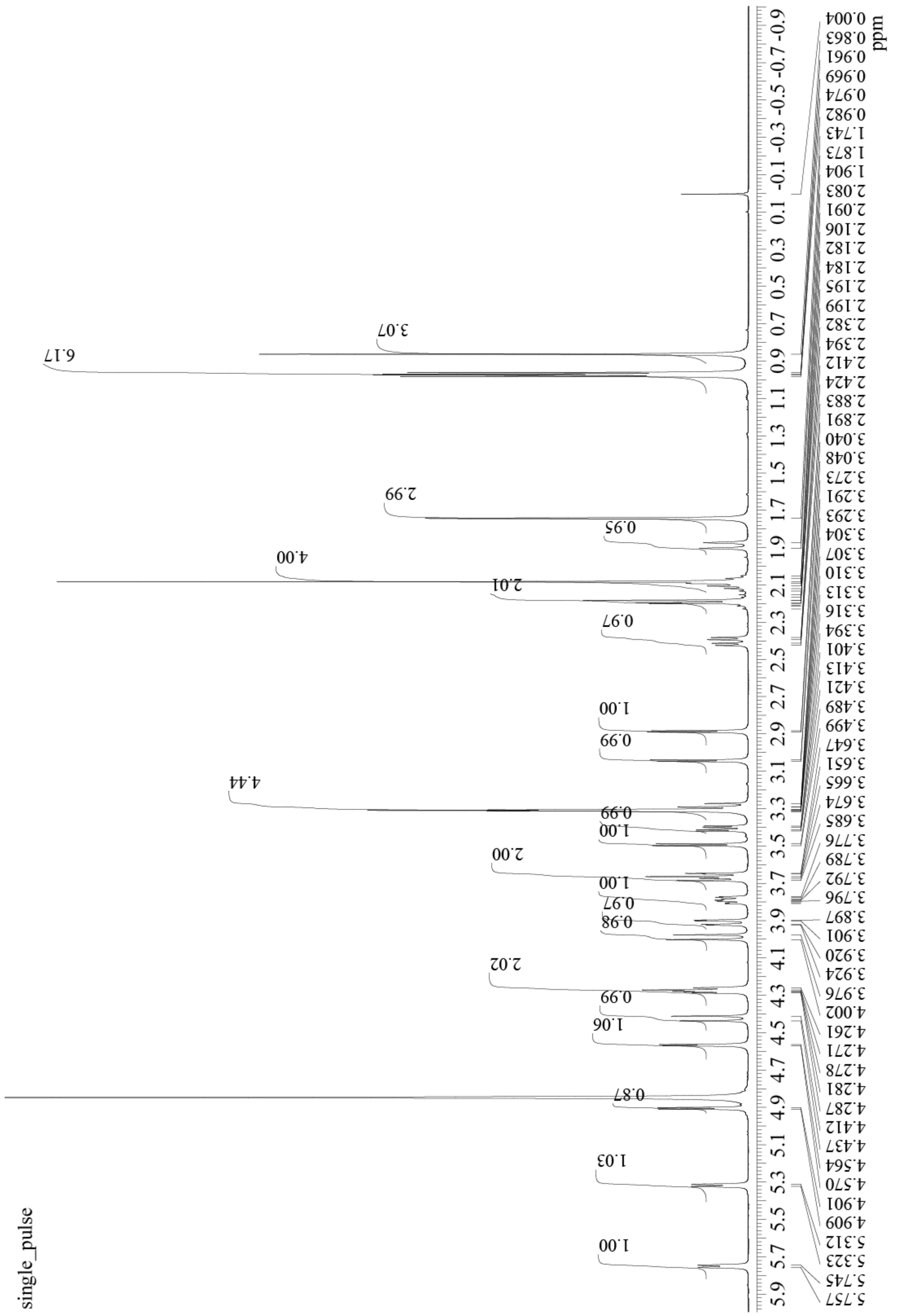

Figure S4-a. ^{1}H NMR spectrum of **3**.

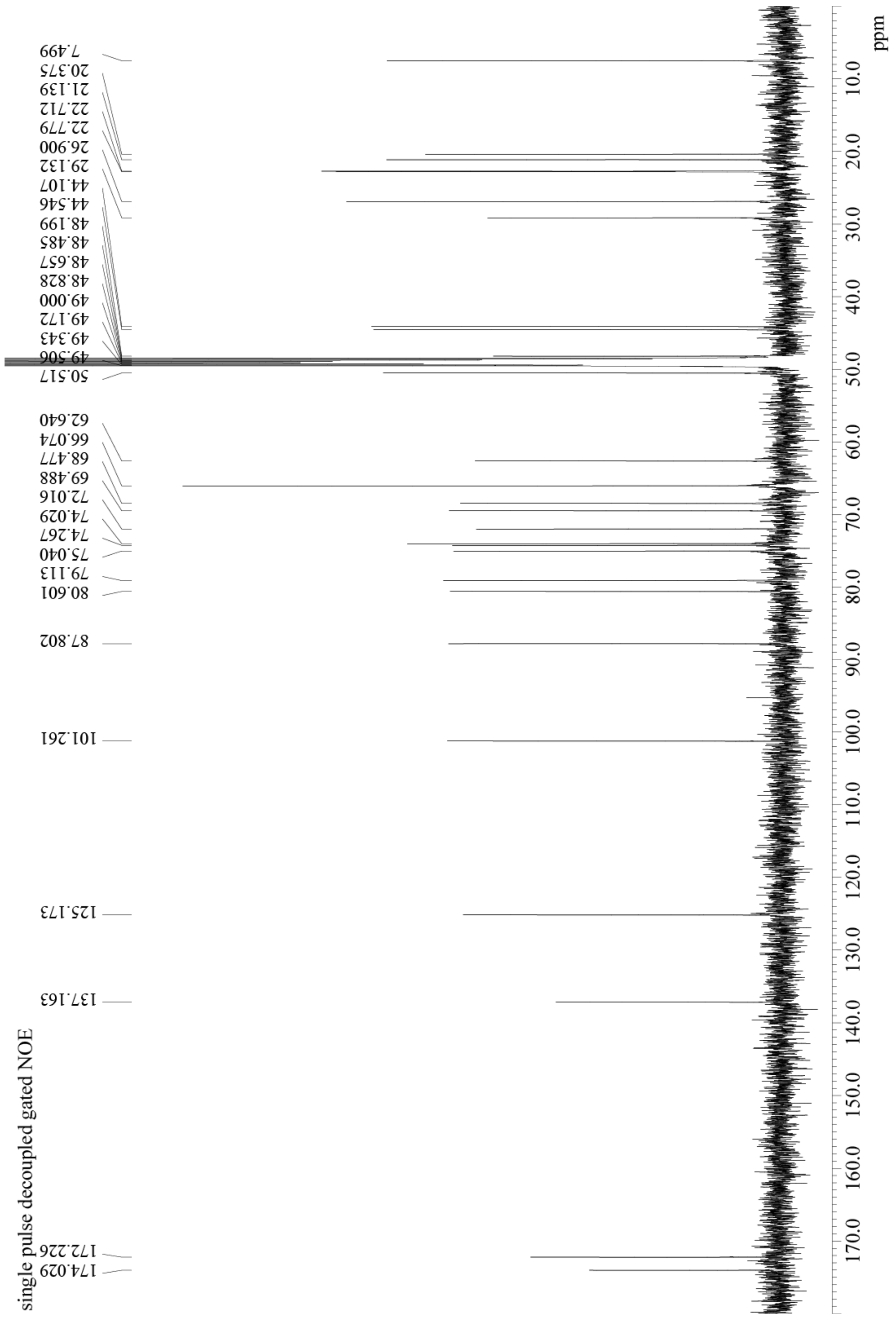

Figure S4-b. ^{13}C NMR spectrum of **3**.

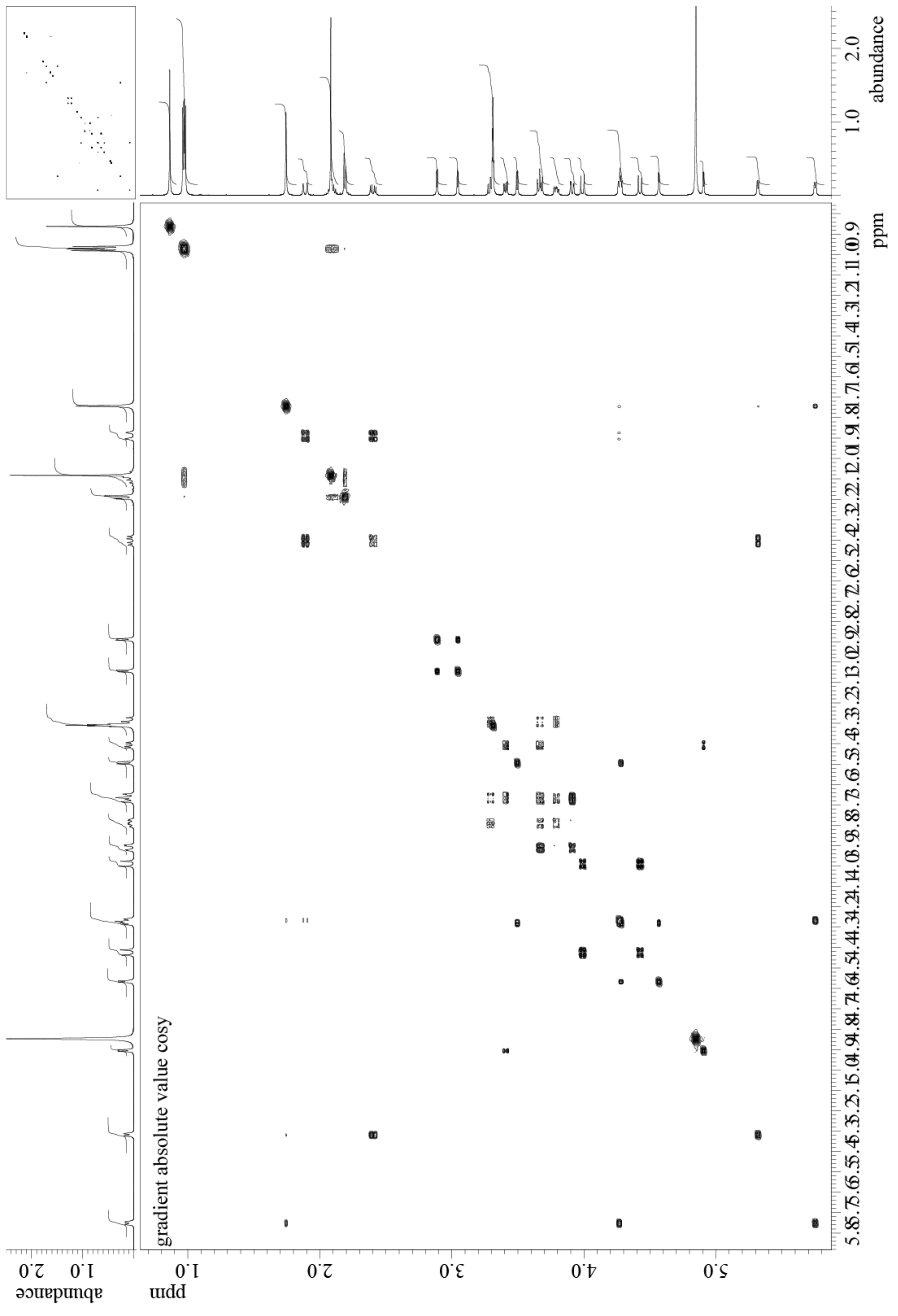

Figure S4-c. Correlation spectroscopy (COSY) spectrum of **3**.

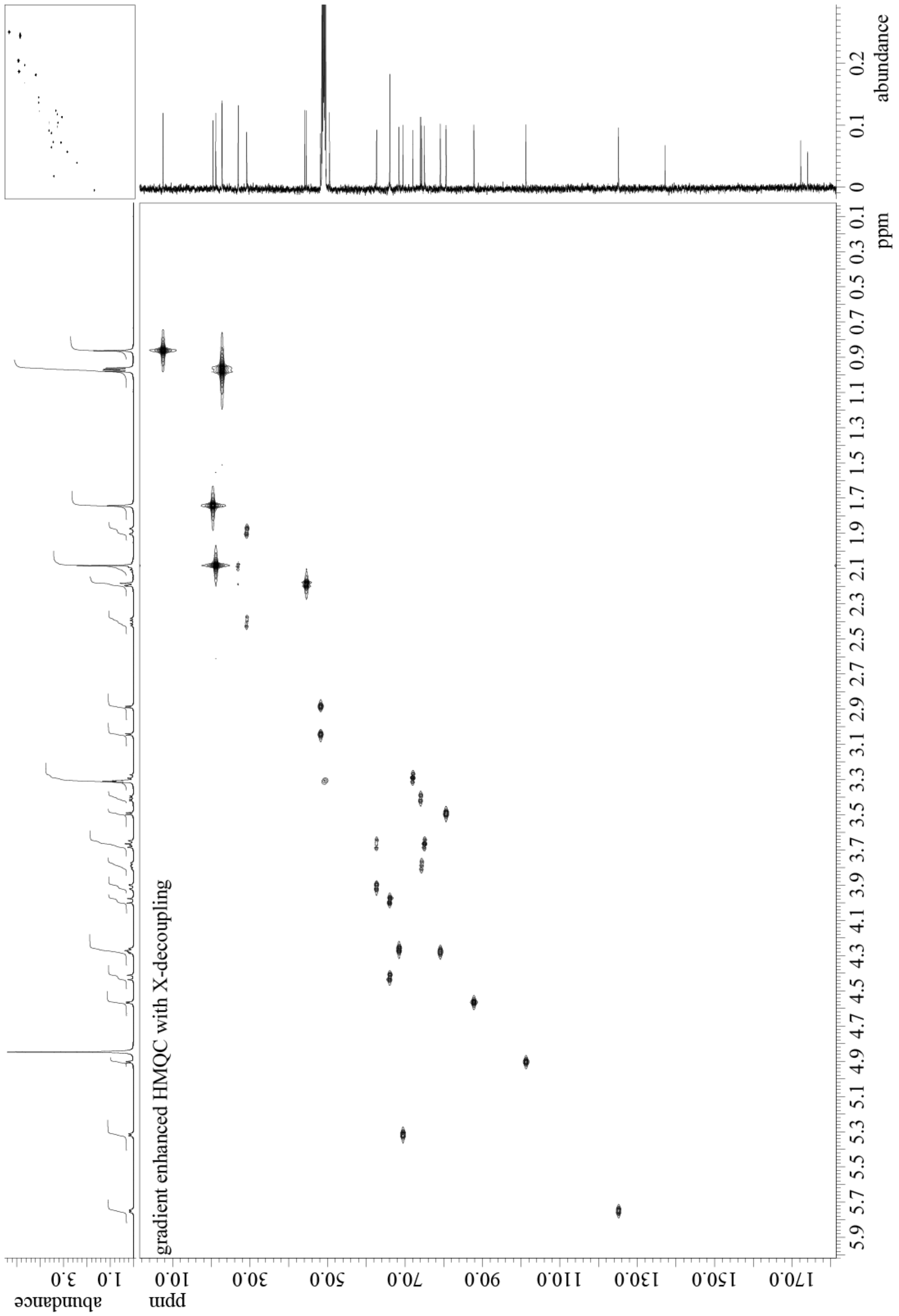

Figure S4-d. Heteronuclear multiple quantum coherence (HMQC) spectrum of **3**.

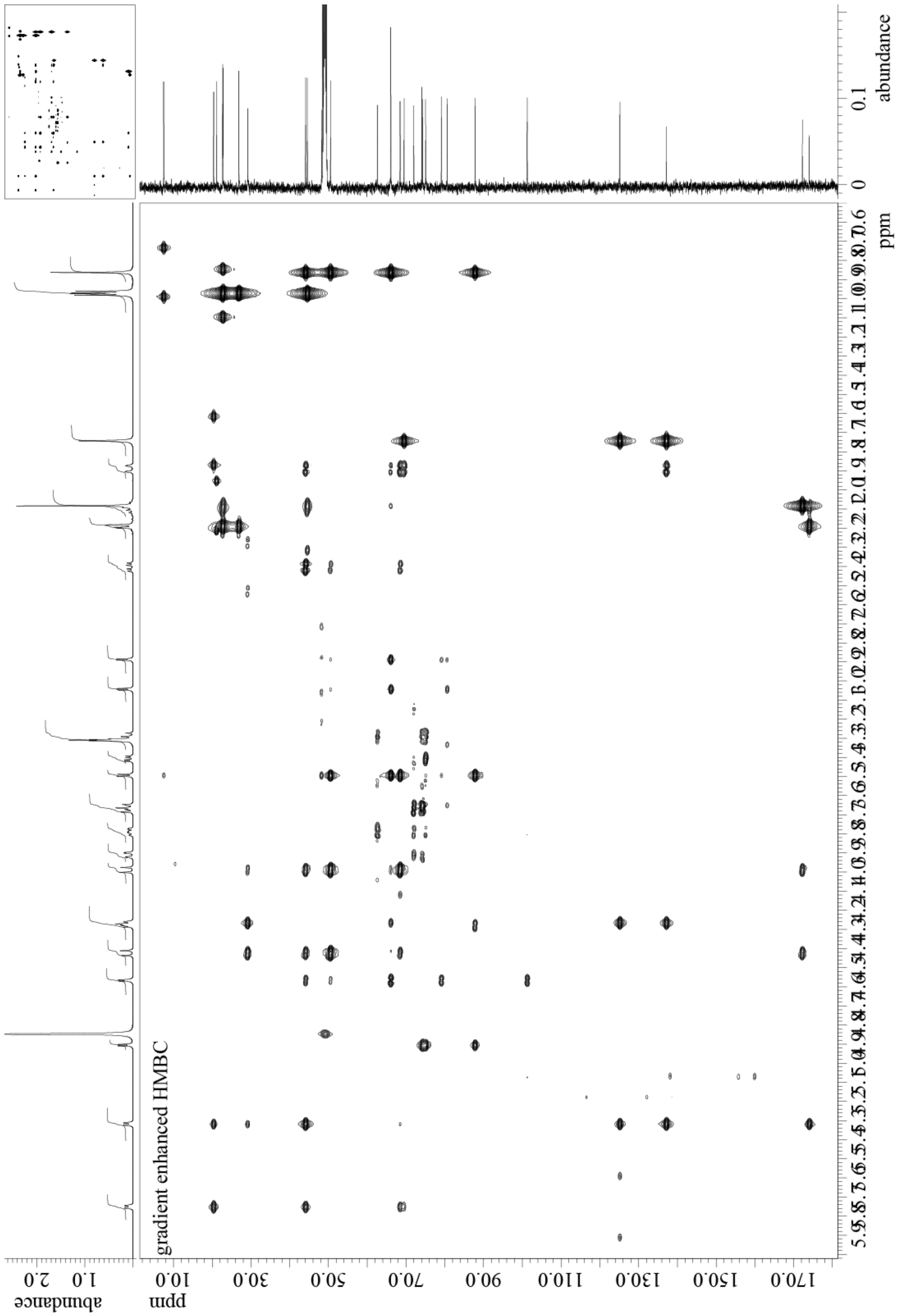

Figure S4-e. Heteronuclear multiple bond coherence (HMBC) spectrum of **3**.

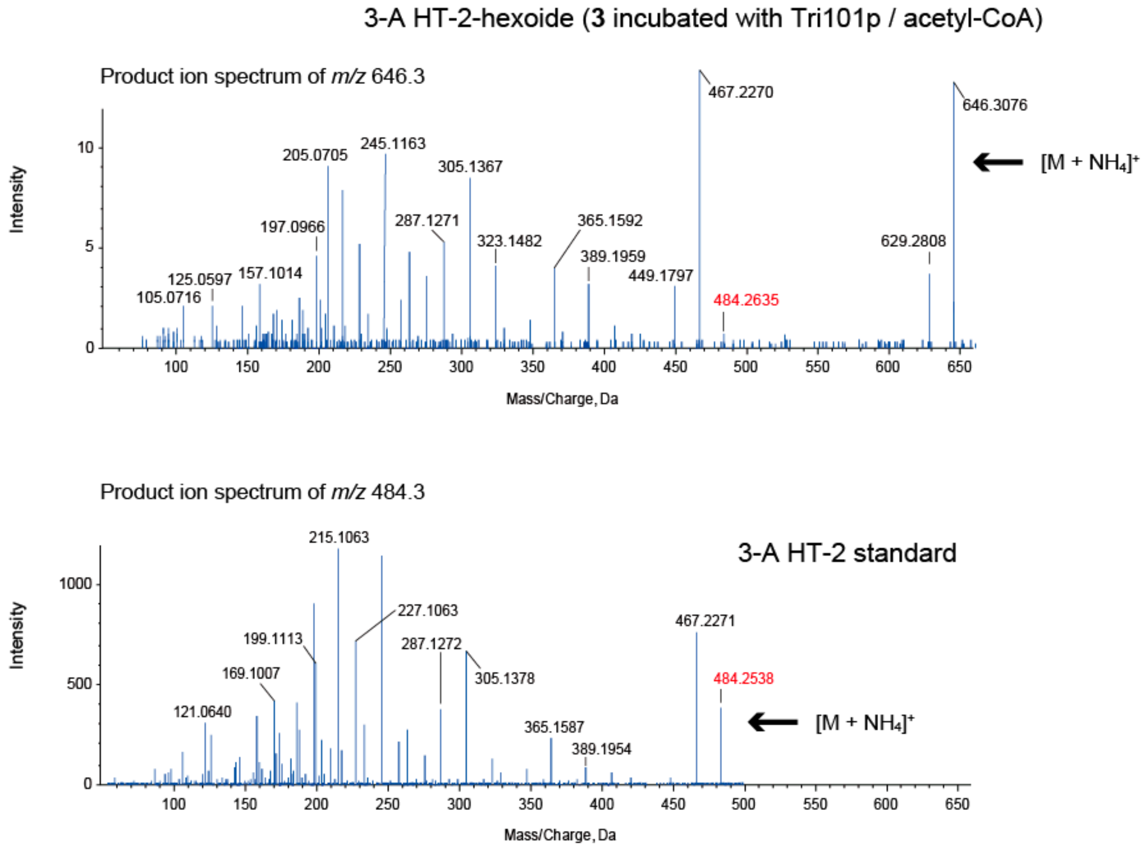

Figure S5. LC-MS/MS analysis of the Tri101p reaction mixture of HT-2-toxin-4-*O*-α-glucopyranoside (HT-2-4-glc) and acetyl CoA. MS/MS spectrum corresponding to *m*/*z* 646.3069 was detected from the mixture after 6 h of incubation (upper penal). MS/MS spectrum of purified 3-acetyl HT-2 toxin (3-A HT-2) standard, detected as [3-A HT-2 + NH_4]$^+$, is shown for comparison.

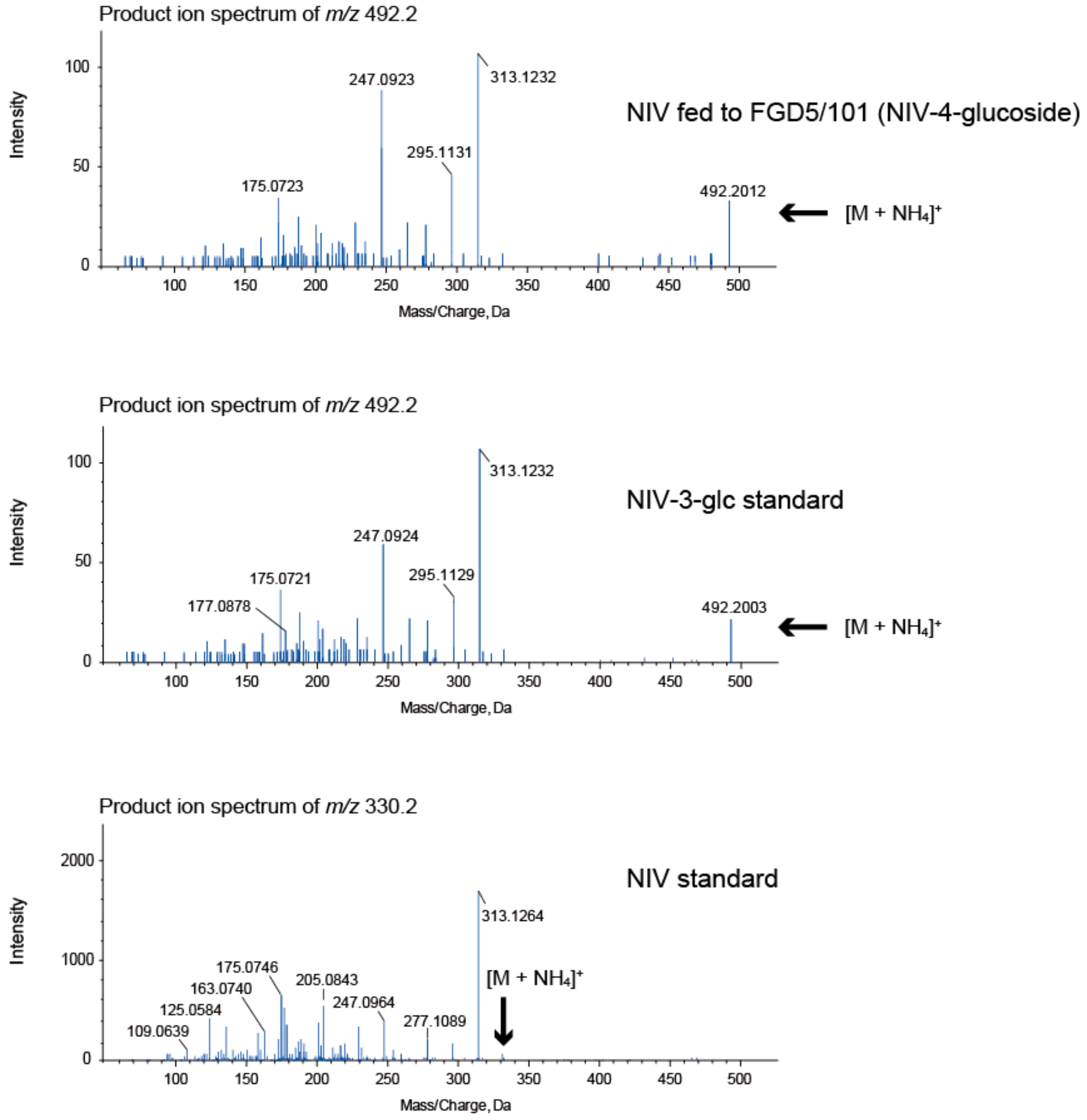

Figure S6. Comparison of MS/MS spectra of putative NIV-4-*O*-glucoside and NIV-3-*O*-β-glucopyranoside (glc) standard. Putative NIV-4-glucoside (upper panel) and NIV-3-glc standard (middle panel) were analyzed by LC-MS/MS in a set of experiment. As a reference, NIV standard was also analyzed under the same condition (lower panel).

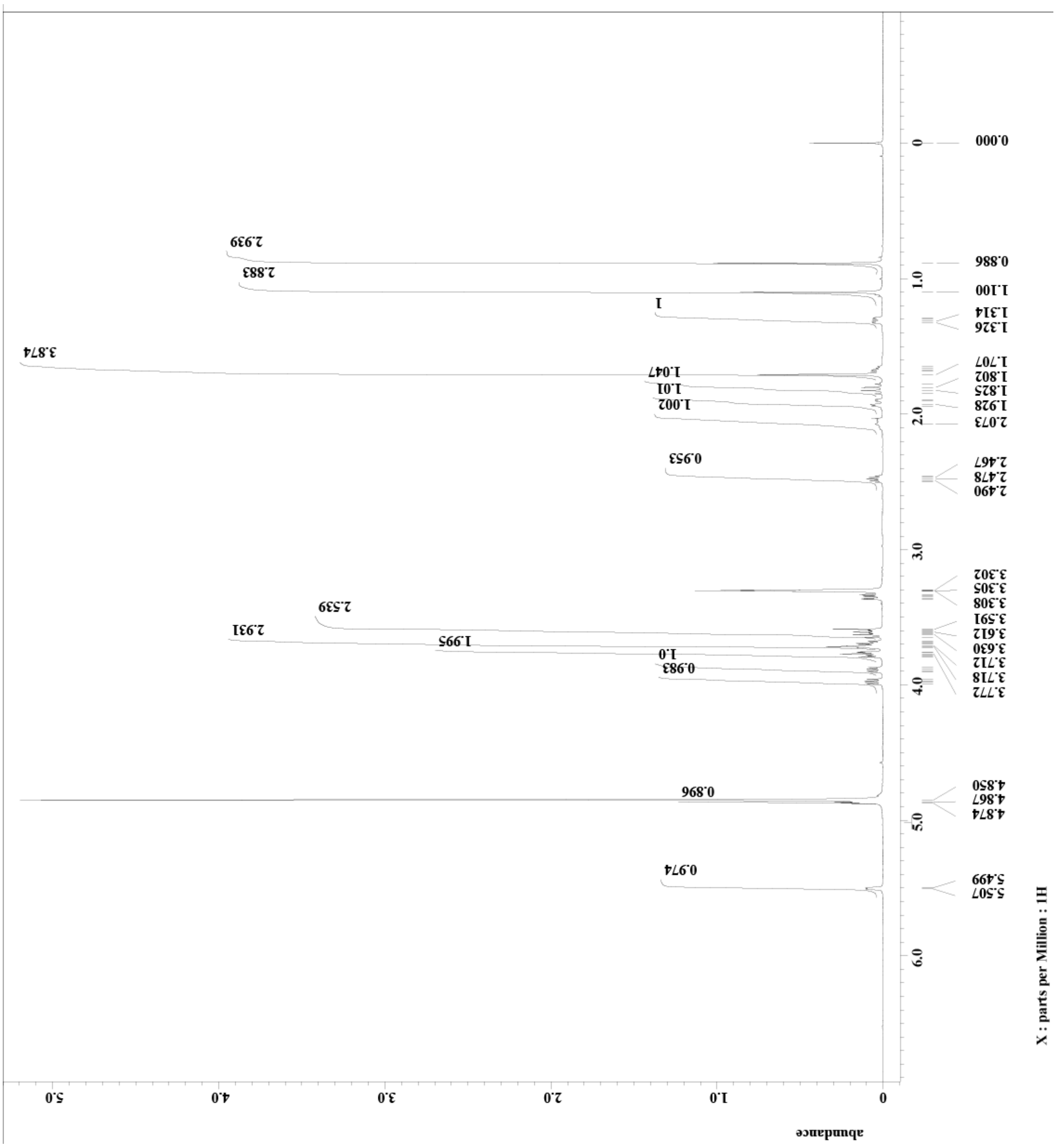

Figure S7-a. ^{1}H NMR spectrum of **4**.

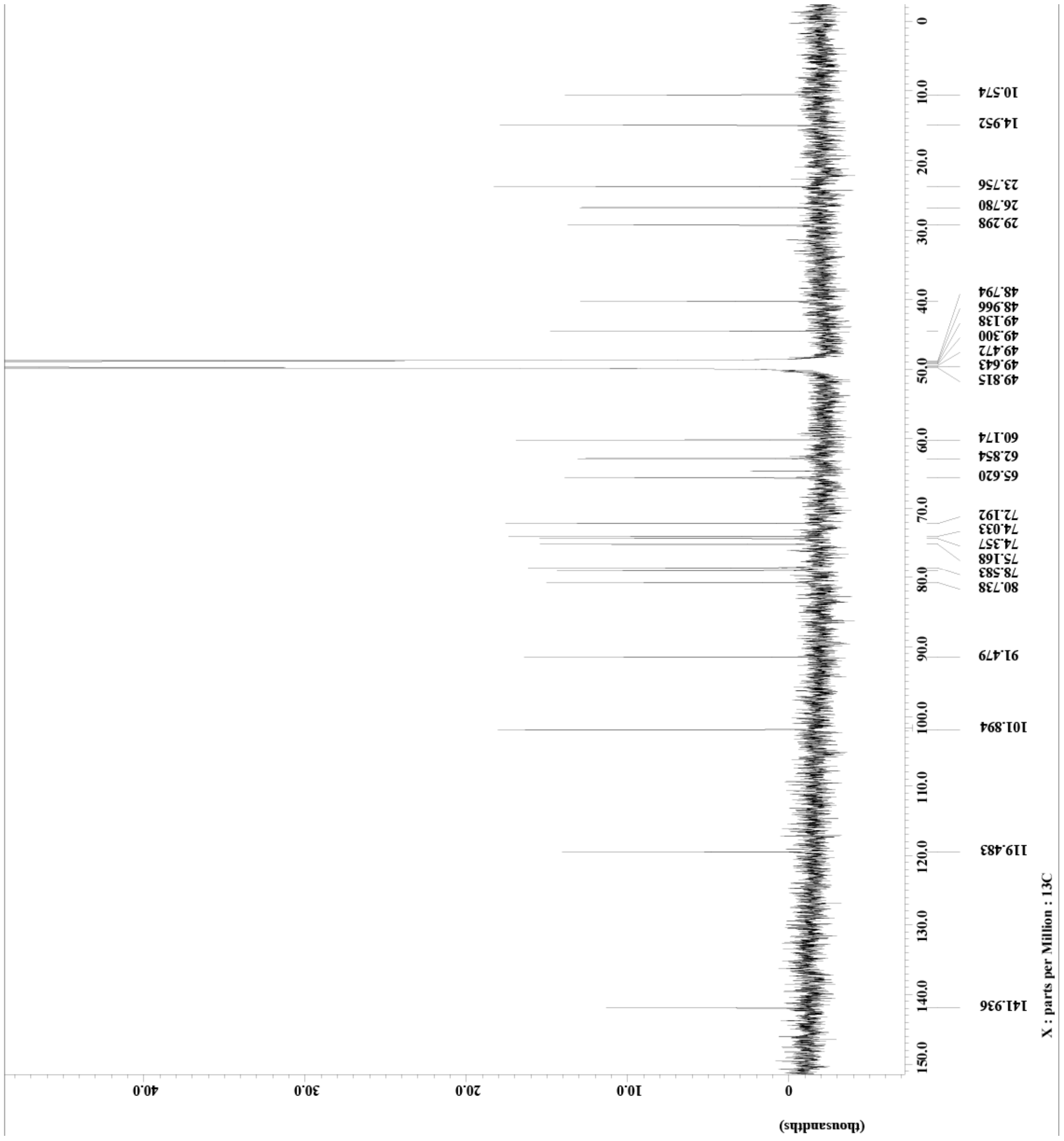

Figure S7-b. ^{13}C NMR spectrum of **4**.

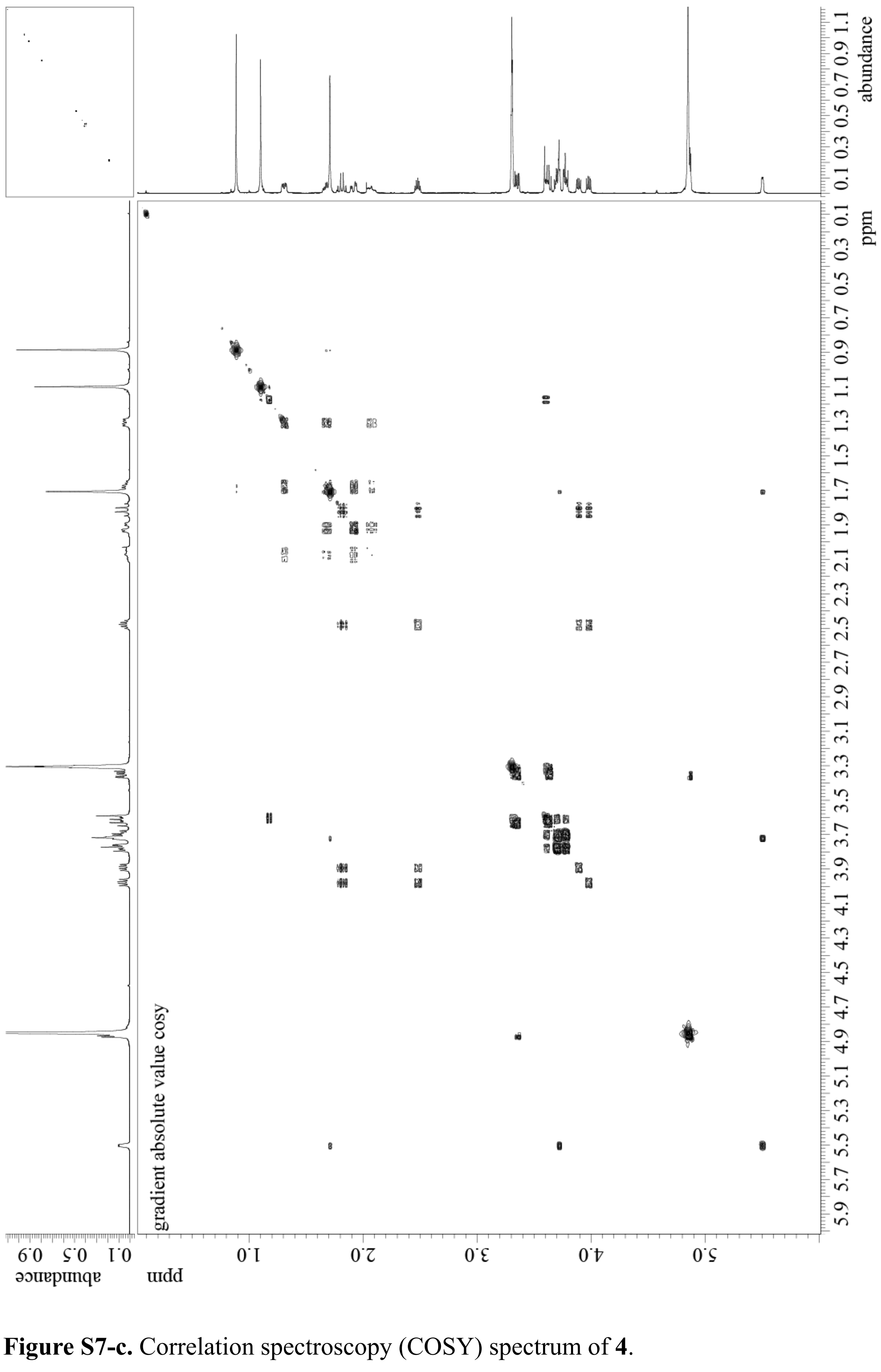

Figure S7-c. Correlation spectroscopy (COSY) spectrum of **4**.

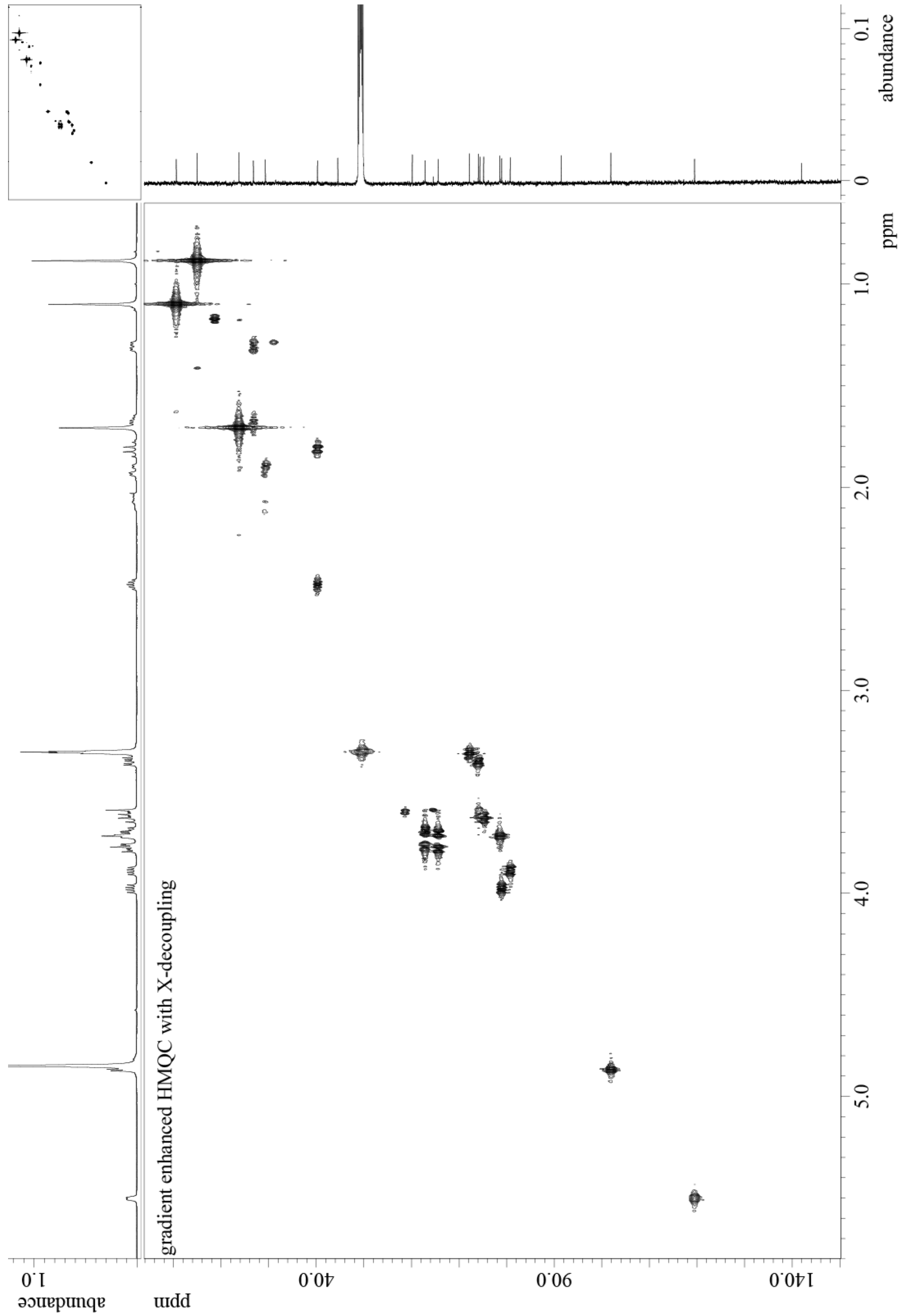

Figure S7-d. Heteronuclear multiple quantum coherence (HMQC) spectrum of **4**.

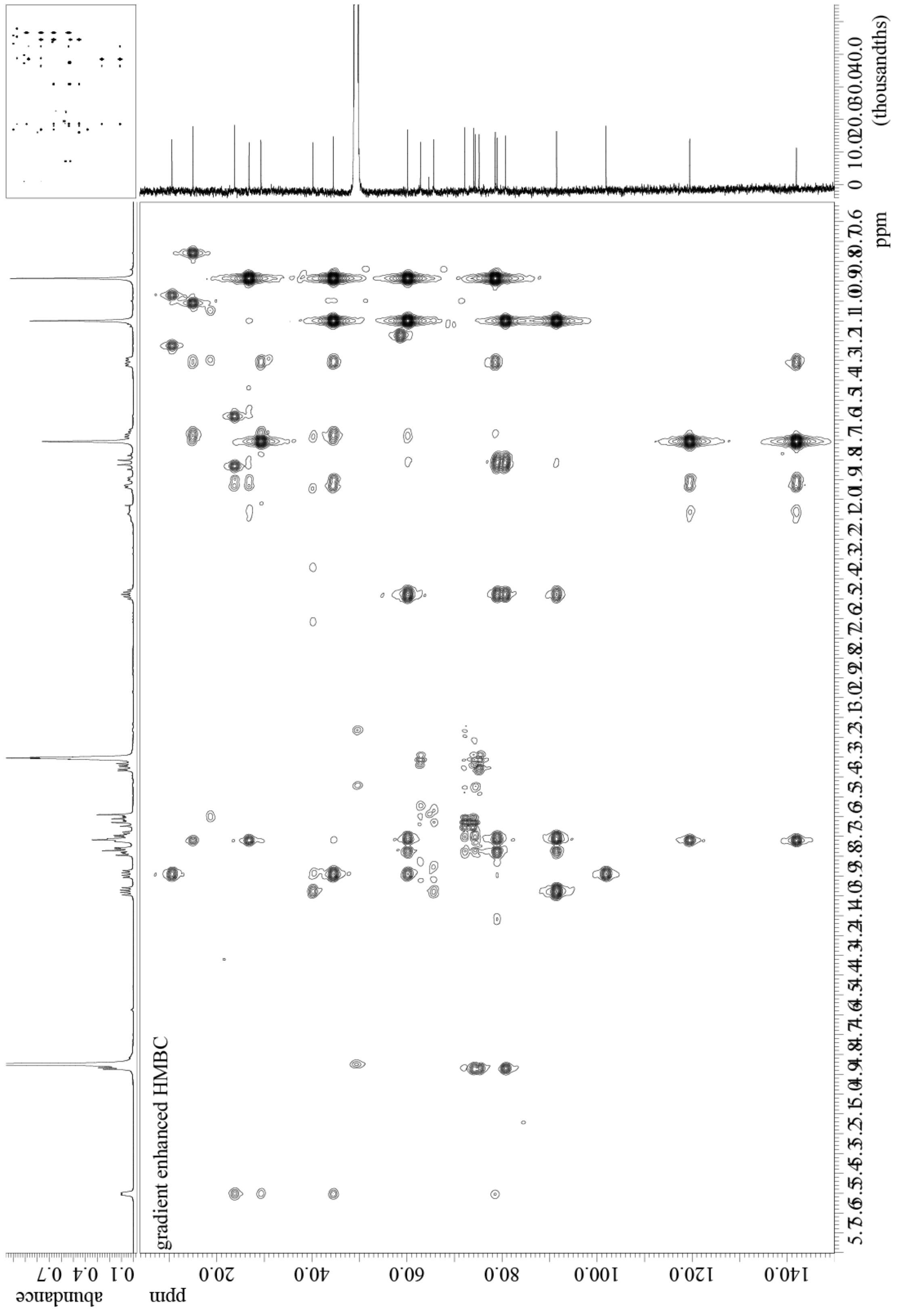

Figure S7-e. Heteronuclear multiple bond coherence (HMBC) spectrum of **4**.

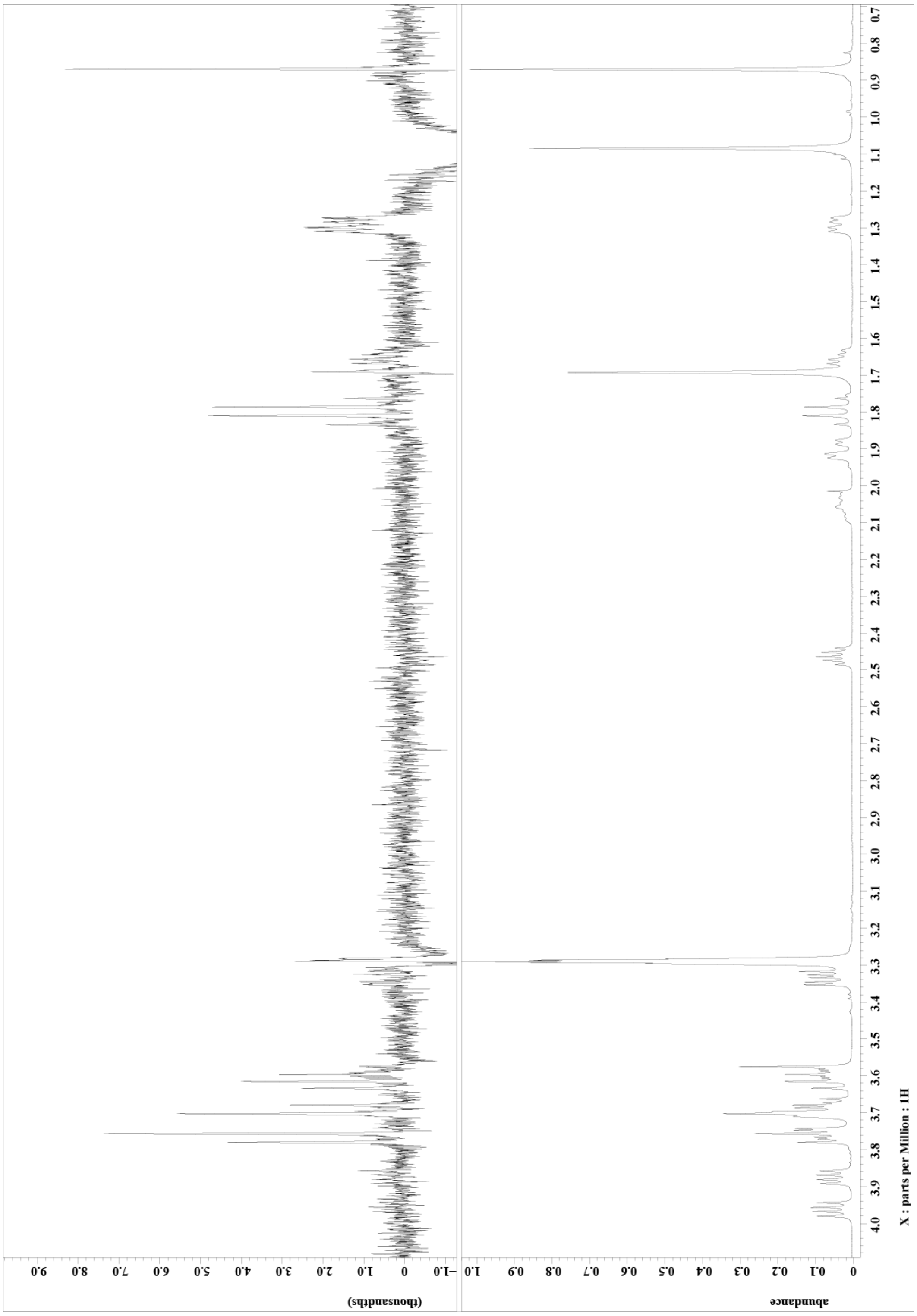

Figure S7-f. 1D-nuclear Overhauser effect (1D-NOE) and ^{1}H NMR spectrum of **4**. The upper is 1D-NOE spectrum obtained by pulse irradiation at 1.10 ppm. The under is ^{1}H-NMR spectrum.

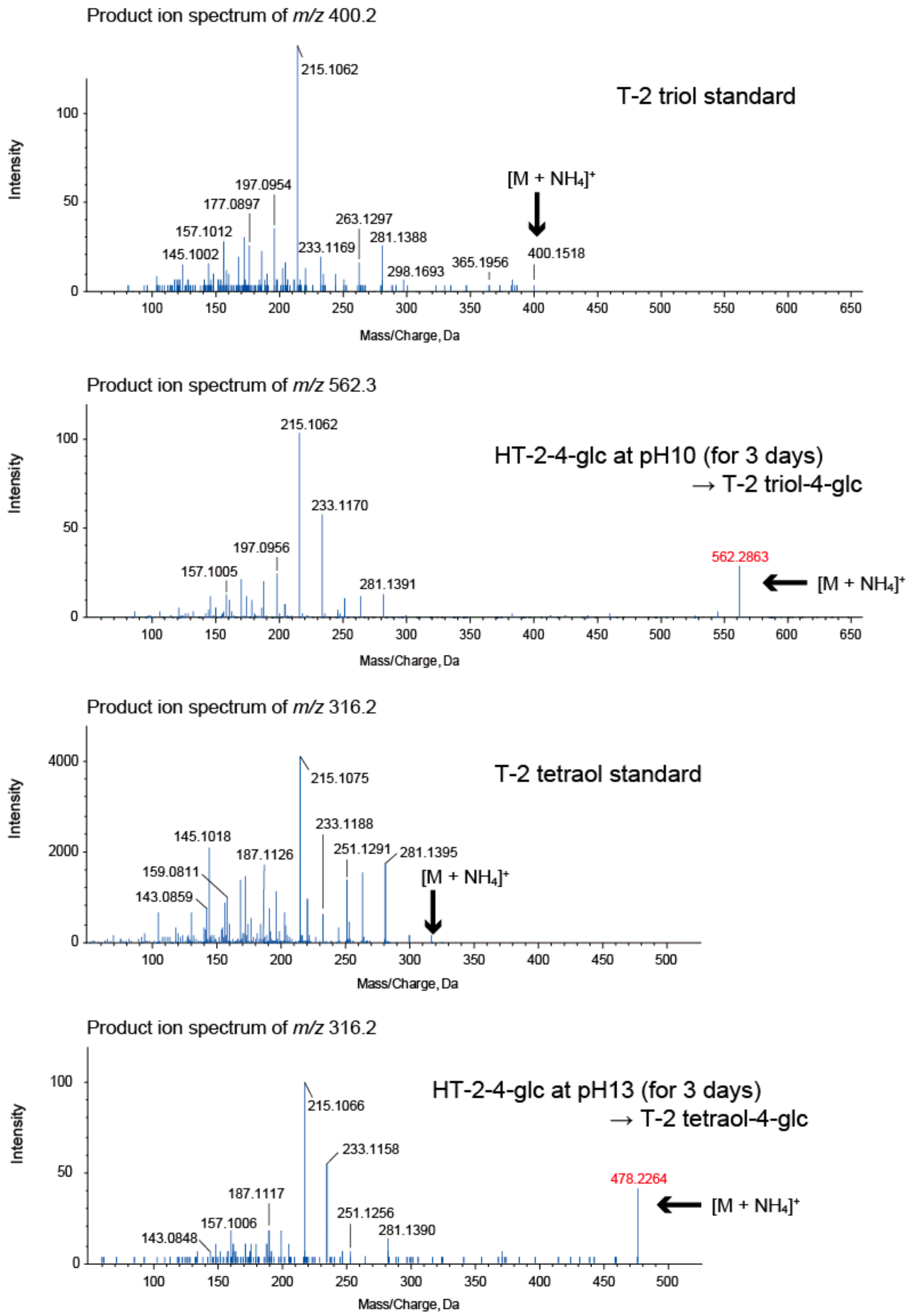

Figure S8. Stability of **3** at alkaline pH. MS/MS spectra of deacetylated products, corresponding to [T-2 triol-hexoside + NH_4]$^+$ (panel 2; pH10 for 3 days) and [T-2 tetraol-hexoside + NH_4]$^+$ (panel 4; pH13 for 3 days), are shown, together with those of their aglycons (panel 1 and 3).
